# Oxyresveratrol-β-cyclodextrin mitigates streptozotocin-induced Alzheimer's model cognitive impairment, histone deacetylase activity in rats: in silico & in vivo studies

**DOI:** 10.1038/s41598-024-57188-7

**Published:** 2024-04-30

**Authors:** Tushar Agarwal, Suman Manandhar, Harish Kumar B, Ademola C. Famurewa, Prasada Chowdari Gurram, Ramya Shri Suggala, Runali Sankhe, Jayesh Mudgal, K. Sreedhara Ranganath Pai

**Affiliations:** 1 Department of Pharmacology, Manipal College of Pharmaceutical Sciences, Manipal Academy of Higher Education (MAHE), Manipal, Karnataka 576104 India; 2 Department of Medical Biochemistry, Faculty of Basic Medical Sciences, College of Medical Sciences, Alex Ekwueme Federal University, Ndufu-Alike, Ikwo, Abakaliki, Ebonyi State Nigeria

**Keywords:** Dementia, Alzheimer’s disease, Oxyresveratrol, Epigenetics, Streptozocin, In silico docking, Computational biology and bioinformatics, Neuroscience, Cognitive neuroscience, Computational neuroscience

## Abstract

Alzheimer’s disease (AD) is associated with cognitive deficits and epigenetic deacetylation that can be modulated by natural products. The role of natural oxyresveratrol-β-cyclodextrin (ORV) on cognition and histone deacetylase activity in AD is unclear. Herein, in-silico docking and molecular dynamics simulation analysis determined that oxyresveratrol potentially targets histone deacetylase-2 (HDAC2). We therefore evaluated the in vivo ameliorative effect of ORV against cognitive deficit, cerebral and hippocampal expression of HDAC in experimental AD rats. Intracerebroventricular injection of STZ (3 mg/kg) induced experimental AD and the rats were treated with low dose (200 mg/kg), high dose (400 mg/kg) of ORV and donepezil (10 mg/kg) for 21 days. The STZ-induced AD caused cognitive and behavioural deficits demonstrated by considerable increases in acetylcholinesterase activity and escape latency compared to sham control. The levels of malondialdehyde (MDA) and HDAC activity were significantly increased in AD disease group comparison to the sham. Interestingly, the ORV reversed the cognitive-behavioural deficit and prominently reduced the MDA and HDAC levels comparable to the effect of the standard drug, donepezil. The findings suggest anti-AD role of ORV via antioxidant effect and inhibition of HDAC in the hippocampal and frontal cortical area of rats for AD.

## Introduction

Alzheimer’s disease (AD) is a chronic type of neurodegenerative dementia among the elderly and affects more than 35 million people worldwide^1^. It has been estimated that the number may surge to reach 65.7 million by 2030^1,2^. The established pathological hallmarks of AD include neuronal death and significant cognitive derangement orchestrated via excessive amyloid β (Aβ) plaque accumulation and intracellular neurofibrillary tangles (NFTs) in the cerebral cortex and hippocampus^3^. It is the Aβ and the NFTs that are etiologically known responsible for the accompanying neuronal loss, cholinergic aberration, oxidative inflammation, and synaptic dysfunction. Thus, therapeutic approaches targeting mechanistic reduction of neuronal deposits of Aβ and NFTs have attracted attention^4^ nevertheless, there is no effective treatment or cure for AD currently.

Epigenetic dysfunction has been associated with the pathophysiology of neurodegenerative diseases (NDD). Modulation of epigenetic enzymes provoke critical role in the development of AD^5^. Histone acetylation and deacetylation are critical epigenetic mechanisms for cognition, and mediated by histone acetyltransferases and histone deacetylases (HDACs), respectively^6^. HDACs are a group of enzymes that play a role in regulating gene expression by modifying the structure of chromatin. HDACs remove acetyl group from lysine residue of histones and non-histone proteins and regulate the transcriptional process for modulation of cellular proliferation, differentiation and development^7^. While HDACs regulate chromatin structure and transcription by removing histone acetyl epigenetic modification, nonhistone deacetylation influences a variety of physiologic activities^8^. Therefore, inhibition of HDAC is attracting attention for therapeutic purposes in NDD. Earlier, the HDAC inhibitors were used to exert anticancer efficacy^7^. However, the neuroprotection of HDAC inhibitors is accumulating in the existing literature^7,9^. A HDAC inhibitor, intended to serve as a multi-target drug against both HDAC and acetylcholine esterase, demonstrated potent action against Aβ-aggregation and selective inhibition of HDAC6^10^. Since histone deacetylases (HDACs) and acetylcholinesterase (AChE) are two separate enzymes involved in different biological processes, there is a direct correlation between their inhibition. Nonetheless, because of their functions in different biological pathways, there are tangential connections between them. Despite being involved in distinct metabolic pathways, AChE and HDACs may occasionally have an indirect impact on one another due to intricate regulatory networks found in cells. For instance, changes in neurotransmitter levels including acetylcholine may have an impact on signalling pathways that control HDAC activity, however there may be a concrete data supporting these relationships^10^. The distribution of acetylcholine receptors at the neuromuscular junction is more effective when HDAC6 is inhibited^11,12^ Inhibitors of HDAC increase the level of histone acetylation, enhancing memory and learning, and impacting the functions of proteins crucial to Alzheimer's disease^13–15^. In animal models of AD, chronic therapy with HDAC inhibitors, valproate, sodium butyrate, or vorinostat results in a remarkable restoration of contextual memory^7,16^. However, potential toxicity and safety of the chemical HDAC inhibitors is a source of concern. Certain studies report neurotoxicity, mutagenicity and DNA damage of a few HDAC inhibitors^7,16,17^.

The current treatment modalities for AD have side effects, hence the need for more safe and effective agents. Natural product HDAC inhibitors are emerging as therapeutic leads against NDD. A number of natural products have been shown as potential natural inhibitors of HDAC^18^. Oxyresveratrol (trans-2,3′,4,5′-tetrahydroxystilbene) is a potent natural antioxidant compound abundant in the mulberry plant^19^. It is shown to inhibit Aβ expression and prevents neuronal cell death via scavenging of reactive oxygen species and inhibition of neuronal β-secretase^20,21^. Recently, Rahman et al. (2021) shows that oxyresveratrol prevents amyloid precursor protein production in cortical astrocytes. Oral oxyresveratrol decreased behaviour deficits, lipid peroxidation, and neuronal death in AD model of mice with Aβ_25–35_ intracerebroventricular injection^22^. However, the oral absorption and bioavailability of oxyresveratrol is low and limited which has largely restricted its clinical use^23^. To overcome this drawback in the current study, we considered a complex, oxyresveratrol-betacyclodextrin (ORV). Cyclodextrins are linked to molecules to form complexes with improved properties without modification of original structure^24^. Literature shows that inclusion complexes of resveratrol and oxyresveratrol with cyclodextrins possess increased water solubility and antioxidant efficacy^24–26^. Herein, we have used in silico-based screening to identify oxyresveratrol against HDAC2 protein, and therefore aimed to explore in vivo the ameliorative effects of ORV against memory deficits, lipid peroxidation, behavioural and HDAC expression in the hippocampus and cortex of STZ-induced AD rat model.

## Materials and methods

### In silico methodology

Maestro software from Schrodinger Inc. was used for all the computational studies in Linux Ubuntu 18.04.1 operating system. All the desired tools such as protein preparation wizard, LigPrep, GLIDE (Grid-based ligand docking with energetics), MMGBSA (Molecular Mechanics-Generalized Born Surface Area) and Desmond were used for the computational study.

### Molecular docking

The crystallographic structure for HDAC2 protein in complex with Suberanilohydroxamic acid PDB ID: 4LXZ of resolution of 1.85 Å was retrieved from protein data bank. The selected protein was pre-processed using the protein preparation wizard of the Schrodinger suite. Water molecules above 3 Å were removed, missing amino acid residues and hydrogen atoms were added to the protein. Later protein was minimized using OPLS3e force field to generate its lowest energy state. For ligand preparation one thousand eight hundred and thirteen (1813) phytochemicals were selected and downloaded from Zinc 15 database. In Ligprep module of Schrodinger suite, OPLS3e force field was used to obtain low energy level 3D structure at ionisation pH of 7.4 ± 0.5. Apart from this pre-processing of ligands includes hydrogen bond addition, optimizing ligand geometry, and generation of tautomer.

GLIDE tool of the Schrodinger suite was used for docking ligands which ranks the ligands based on their dock scores. Further, using receptor grid generation tool a grid of 20 Å was generated over the amino residue of the ligand-bound active site. The ligands were subjected to varied scoring functions such as HTVS (high throughput screening), SP (standard precision), XP (extreme precision). Initially the compounds were screened in HTVS and further screened based on their dock scores^27^. Further, the compounds were screened using SP and XP as it removes any false-positive result.

### Free binding energy calculation

To evaluate ligand binding energy with HDAC protein prime module from Schrodinger was employed. MM-GBSA was performed to calculate the free binding energy of top 10 molecules selected from XP docked drugs based on docking score and interaction with the amino acid. To calculate binding energy VGSB, solvation model and water as solvent were used.

### Induced-fit docking (IFD)

Induced-fit docking module was used in Schrodinger suite to perform IFD. Based on XP and dock score, and free binding energy 5 molecules were selected for IFD docking. During IFD calculation Vander Waal’s scaling for the receptor and ligand was maintained to 0.50, and 20 poses were generated. Out of the generated poses, one best pose was selected for MD simulation study^28^.

### Molecular dynamic simulation

Although, Docking gives an insight on how the drug interacts with receptor, it will not mimic the biological environment in which the drug will interact with the protein; hence, MD studies were performed^29^. XP dock score, free binding energy, IFD score and important amino acid residue^30^ involved in interaction with HDAC2 protein, were considered to select 2 molecules for MD simulation studies at 100 ns using NPT (isobaric and isothermal ensemble) at 300 K and 1.01325 bar pressure. MD system was built including the XP docked ligand in the 4LXZ protein in a predefined simple point charge (SPC) water model in an orthorhombic boundary of 10*10*10 Å dimension. The protein–ligand complex was neutralized by the adding necessary sodium and chloride ions. The built system was minimised then 100 ns simulation study was performed. Results of MD simulation study was analysed by generating the simulation interaction diagram.

### In-vivo methodology

#### Animals

For the current investigation, male Sprague Dawley rats weighing 250–300 g were procured from the inbred strains of Central Animal Research Facility, Manipal Academy of Higher Education after obtaining Ethical Clearance from the Institution’s Research Ethics Committee (IAEC/KMC/127/2020). The animals were caged in propylene cages, 3 animals per cage maintained under the optimal temperature with free access to chow diet and potable water ad libitum (1 week acclimatization period). The animal handling and care were done in accordance with guidelines of the Institution’s Research Ethics Committee.

#### Chemicals

Streptozotocin, 2-thiobarbituric acid (TBA), disodium hydrogen phosphate anhydrous, sodium dihydrogen phosphate anhydrous, and trichloroacetic acid were purchased from Sigma-Aldrich (Sigma-Aldrich Co., St Louis, MO, USA). ORV were provided by Sira Naturals, Kerala, India as a gift sample. HDAC fluorometric activity assay kit was purchased from Cayman Chemicals (Ann Arbor, Michigan, USA). All other chemicals used in this study were of analytical grade obtained from Sisco Research Laboratories (SRL), India.

#### Experimental design

##### Intracerebroventricular (ICV) injection of STZ

The surgery was performed under sterile conditions using stereotactic frame from NeuroStar with stereo drive software. Rats were anesthetized using ketamine (90 mg/kg) and xylazine (10 mg/kg) administered intraperitoneally. The animal’s head was shaved, an incision was made along midline of scalp to expose bregma. Bregma was used as a reference point for the software. 5μL of Artificial cerebrospinal fluid (ACSF) (composition:147 mM NaCl, 2.9 mM KCl, 1.6 mM MgCl_2_, 1.7 mM CaCl2, and 2.2 mM dextrose) administration to sham control group and STZ (3 mg/kg, 5 µl) administration was done bilaterally at the coordinates 0.8 mm AP,1.8 mm ML, 3.6 mm DV^31^ to all other group of rats using Hamilton micro syringe at infusion rate of 1 µl/min. After completion of the infusion, it was ensured that the syringe is not disturbed for at least 2 min to prevent the backflow. The rats were sutured, antiseptic was applied, and animals were provided with palliative care post-surgery. Similar procedures were repeated on day 3 for administration of STZ in the same coordinates.

#### Treatment regimen

The animals were randomly divided into five groups with eight animals in each group based on the pre-treatment behavioural test (Morris’s water test) performed before ICV procedures. The administration to rats is as follows:

*Group 1* (Sham control): ICV artificial cerebrospinal fluid (ACSF), 5 μL per ventricle on day 1 and day 3.

*Group 2* (STZ AD control): STZ (3 mg/kg, 5 µl), ICV per ventricle on day 1 and day 3.

*Group 3* (STZ + ORB 200 mg/kg): STZ (3 mg/kg, 5 µl), ICV per ventricle on day 1 and day 3 + ORB (low dose of 200 mg/kg body weight) administration for 21 consecutive days.

*Group 4* (STZ + ORB 400 mg/kg): STZ (3 mg/kg, 5 µl), ICV per ventricle on day 1 and day 3 + ORB (high dose of 400 mg/kg body weight) administration for 21 consecutive days.

*Group 5* (Donepezil): STZ (3 mg/kg, 5 µl), ICV per ventricle on day 1 and day 3 + Donepezil at 10 mg/kg p.o administration for 21 consecutive days^32^.

**Figure 1:**
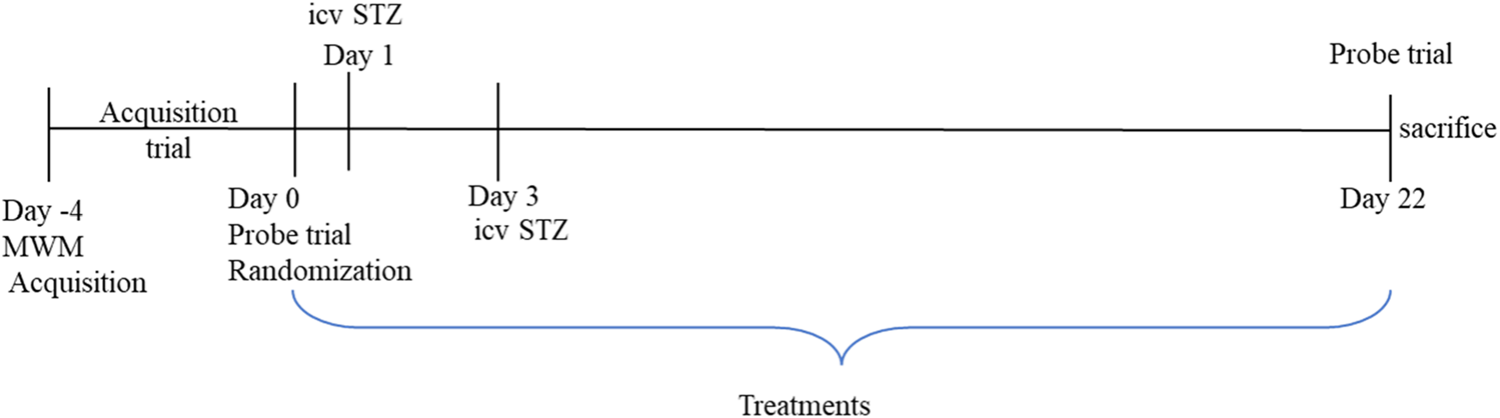
Time schedule used for the study.

Time schedule that we have followed during study has been represented in Fig. 1. Behavioural MWM study was performed on day 22. Animals were euthanised using high dose of anesthetic (thiopental sodium, 80 mg/kg, i.p.) and brains were isolated followed by separation of hippocampus and frontal cortex and preserved in − 80 °C freezer till further biochemical analysis. The brain samples were homogenised in 0.1 M ice-cold phosphate buffer (pH 7.4) for biochemical analyses in nuclear extraction buffer (20 mM HEPES, 10 mM KCl, 2 mM MgCl_2_, 1 mM EDTA, 1 mM DTT, 1 mM EGTA) for HDAC enzyme estimation by fluorimetry.

#### Behavioural study

Morris water maze (MWM) was employed to access spatial memory and learning in the rodents. MWM consists of a pool with 150 cm diameter and 40 cm depth filled with water 2 cm above the platform. The water was turned opaque using milk powder so that the animal was not able to locate the surface also to avoid false positive. Before the commencement of the experiment all the animals were shifted to the designated room and were acclimatized for a day. ANYMAZE software was used to detect and record the data. The whole apparatus was divided into 4 quadrants A, B, C, D and a platform, named island zone of 10 cm. The platform was kept in D quadrants just beneath the water surface. Apart from these various external cues of various geometric shapes were prepared and were attached to specific points in the pool. Fresh water was filled every day before performing the experiment. To record the data a camera was fixed above the pool and the animal movement was tracked, low light intensity was maintained throughout the Experiment. Each animal was trained for 4 trials to reach to the platform with aid of external cues for 4 consecutive days by gently placing the animals to the different quadrants with head facing to the wall. The animals were monitored for a period of 60 s. In case the animal was not able to find the platform it was guided to the platform manually and allowed to stay for minimum 20 s to recognize the cues. Once the trial was done it was dried gently using a clean towel and was gently placed back in cage.

The probe trial was performed on day 5 to evaluate whether the animals can reach the platform. In this trial the platform was removed, and the animals were placed gently on the opposite side of the platform and the data was recorded for 60 s. Using ANYMAZE software various parameters were evaluated such as average speed, D time, escape latency, path efficiency, the number of times animal crosses the platform area was recorded.

#### Estimation of acetylcholinesterase activity

Modified Ellman’s method was used to estimate the activity of acetylcholinesterase (AChE) in the isolated brain samples. Acetylthiocholine is used as a substrate, that is lysed by AChE to give thiocholine and acetate. The sulfahydryl group of thiocholine reacts with DTNB (5,5-Dithio bis (2-nitrobenzoic acid) to yield yellow color^31,33^. The intensity of this yellow color is measured by UV spectrophotometer, the activity corresponds to the intensity of the color produced. Briefly, 25 µl of tissue homogenate was added to 130 µl of phosphate saline buffer (pH 7.4). To this 20 µl of 0.5 mM DTNB and 1 µl of 20 mM acetylthiocholine was added. The absorbance of the generated color intensity was recorded for a period of 4 min with interval at 60 s.

#### Estimation of lipid peroxidation

Lipid peroxidation (LPO) level in the brain sample was measured as per the method described earlier^31,34^. Equal volume of brain homogenate (100 µl each) and TBA-TCL reagent were mixed, heated in water bath maintained at 90 °C for 10 min. The development of pink color was observed which was centrifuged at 5000 rpm for 5 min at 4 °C to collect the supernatant. Malonaldehyde (MDA) concentration was measured spectroscopically at 532 nm. Estimation of the total protein was performed for all the samples using QPRO–BCA Protein Assay Kit (Cyanagen, Italy), as per the manufacturer’s instructions.

#### Estimation of HDAC expression level

The brain samples were homogenized using nuclear extraction buffer, centrifuged and supernatant was collected. HDAC activity assay (Cayman Chemical Co., MI, USA; #10011563) was performed according to the manufacture’s protocol carried out in triplicates. The data are presented as fluorescence units.

#### Histopathology

The isolated brain samples were put in 10% formalin solution for fixation followed by paraffin embedding, sectioning to a 5 µm thick section. The sections were stained using thioflavin stain for amyloid staining, hematoxylin, and eosin dye for histopathological evaluation.

### Statistical analysis

The data obtained in the in vivo study were analyzed by applying ANOVA followed by Tukey’s multiple comparison post hoc test using GraphPad prism 8.2.0. All the data are represented as Mean ± SEM. *p* < 0.05 was considered as statistically significant.

### Ethical approval

Animals used in the current study was procured from the inbred strains of Central Animal Research Facility, Manipal Academy of Higher Education after obtaining Ethical Clearance from the Institution’s Research Ethics Committee (IAEC/KMC/127/2020). The study is reported in accordance with ARRIVE guidelines.

## Results

### Protein preparation

**Figure 2:**
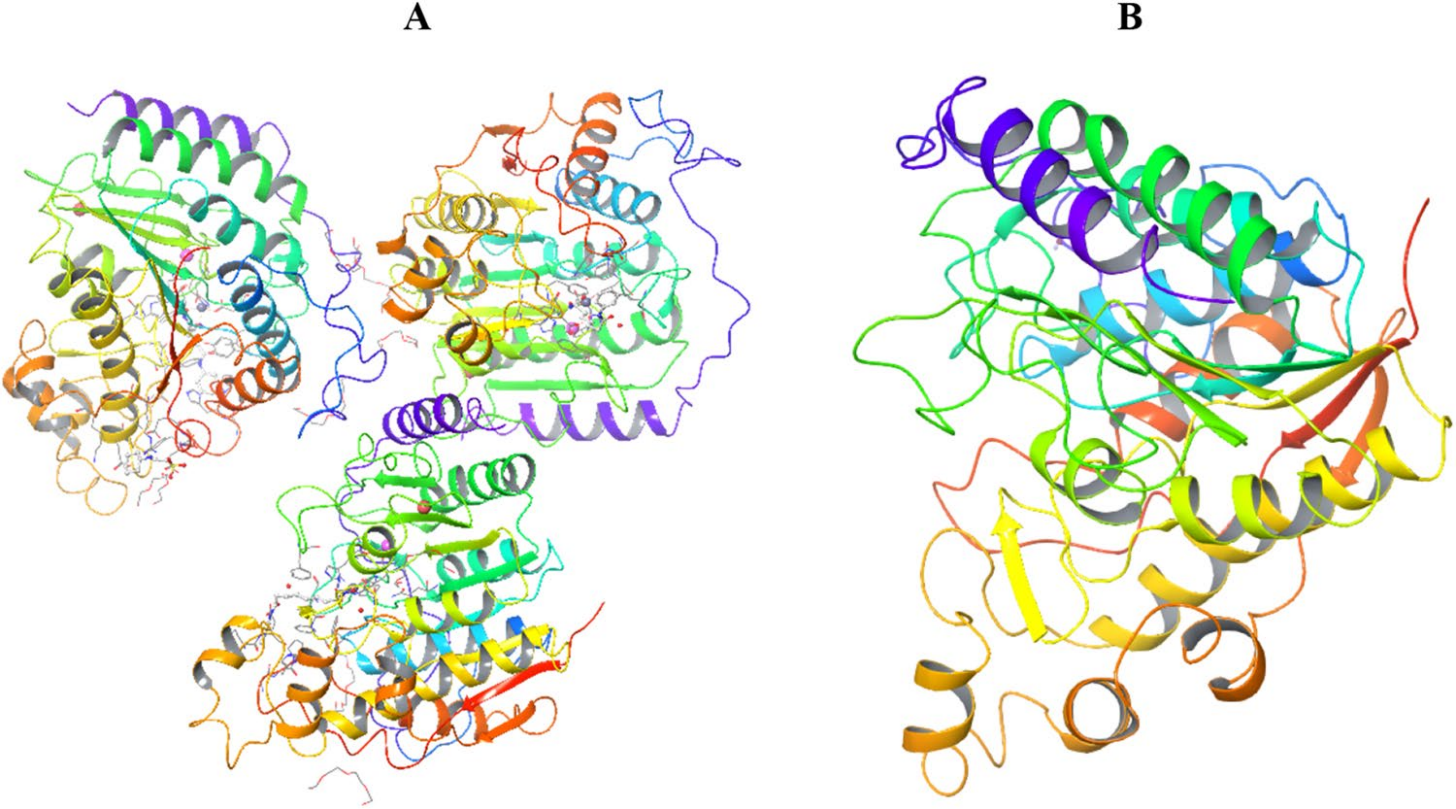
HDAC2 protein (PDB ID: 4LXZ). (**A**) Protein before processing in protein preparation wizard; (**B**) Protein after processing in protein preparation wizard.

HDAC2 protein was prepared at neutral pH 7.4 ± 0.5. The protein consists of three chains namely A, B and C with the chain length of 368 amino acids apart from these it consists of three ions Na, Zn, and Ca. In the minimization process of protein, the B and C chain were removed as can be observed in the Fig. 2. The uniport sequence Q92769 coincides with chain A at residues number 8 to 376^35^ Zinc binding plays a crucial role in the structural integrity of HDAC inhibitor; hence it was conserved, and the remaining two ions were removed. In the protein, the co-crystallized ligand SAHA at the active site is interacting with key amino acid residues HIS145, TYR308, ASP104, HIS186. The receptor grid of specific dimensions was generated around the catalytic site using ‘Receptor grid generation’ and docking studies were performed.

### Virtual molecular docking and binding free energy calculation

Initially the docking protocol was set by re-docking the co-crystallized ligand SAHA in active site. RMSD value was calculated to evaluate the precision in pose coordinated between redocked and native ligand. In the active site the redocked ligand SAHA made crucial interaction by forming a metal coordination complex with Zn which is important for a HDAC inhibitor apart from these it also made, hydrogen bond with HIS145, TYR308, ASP104, ASP181. Taking these results into consideration the further docking protocol was set similarly. All 1813 Ligands from zinc 15 database were docked on the grid generated around the active site. Initially all the ligands were subjected to HTVS docking. HTVS reduces the number of intermediate conformations throughout the docking funnel and reduces the thoroughness of the final torsional refinement and sampling. Hence, 756 ligands were filtered based on a cut off dock score of − 6 kcal/mol and were again analyzed using SP docking mode. Once SP docking was finished a total of 374 potential compounds were selected for XP docking. Finally, based on the dock score and important interacting amino acid residues a total of 10 ligands were sorted out for free energy calculation and IFD analysis. Selected molecules showed docking score ranging from − 10.377 to − 8.901 kcal/mol and binding free energy of range − 48.65 to − 18.65 kcal/mol as tabulated in Table 1.

**Table 1:** Dock score, MM-GBSA dg bind, interacting amino acid residues and 2D interacting diagram of top 10 molecules selected from XP docking.

Ligand zinc database ID	Dock score	MMGBSA dg bind (Kcal/mol)	Interacting amino acid residues	2D interaction diagram
ZINC000000899161	− 10.377	− 32.25	**H-Bond**:- HIS146, HIS183, GLY143	
			**Pi-Pi stacking:**- HIS 183, PHE210	
			**Metal Coordination**:- Zn401	
ZINC000040441974	− 10.24	− 48.65	**H-Bond**: TYR308, GLU208	
			**Pi-Pi Cation**: Phe155	
			**Metal Coordination:** Zn401	
ZINC000040441976	− 9.51	− 35.27	**H-Bond**:- GLU103,	
			**Metal Coordination:**- Zn401	
ZINC000006484604	− 9.490	− 18.65	**H-Bond:**- HIS146, LEU276	
			**Pi-Pi Stacking**:- HIS183, PHE155, HIS146, TYR209	
			**Metal Coordination:- **Zn401	
ZINC000002020259	− 9.49	− 35.88	**H Bond:- **HIS146, TYR308,	
			**Pi cation**:-PHE155, PHE210, HIS183	
			**Metal Coordination**:- ZN401	
ZINC000000056474	− 9.38	− 29.84	**H-Bond:- **GLU103, TYR308, HIS146	
			**Metal coordination**:- ZN401	
			**Pi-Pi stacking**:- PHE210	
ZINC000000113415	− 9.178	− 47.12	**H-Bond**- ASP104	
			**Pi-Pi Stacking**:- HIS146, HIS183, PHE155	
			**Metal Coordination**:- Zn401	
ZINC000086028690	− 9.062	− 40.82	**H-Bond**:- GLU103, TYR308	
			**Metal Coordination:- **Zn401	
ZINC000000004164	-9.072	-37.71	**H-Bond:- **GLY154, HIS 146	
			**PI-PI Stacking**:- TY209, PHE210, HIS183	
			**Metal Coordination**:- Zn401	
ZINC000019632628	− 8.901	− 42.85	**H bond:- **HIS146	
			**Metal Coordination:-** ZN401	

### Induced-fit docking (IFD)

After the XP docking and analyzing free binding energy score 5 ligands showing good interactions, and binding energy were taken for IFD. IFD, a flexible docking protocol was used to generate the most stable pose of the ligand with respect to target protein. IFD generates 20 different poses of each ligand, ZINC000000899161 shows same non-bonding interactions as seen in XP docking, although there were more pipi stacking interactions with PHE210, HIS183, PHE155 after IFD. New interactions were seen with ZINC000006484604, hydrogen bond interaction LEU276, HIS 146, and Pi-Pi stacking TYR209, HIS146, HIS183, PHE210. The most stable binding pose was selected as the starting binding pose for further MD simulation study.

### MD simulation

MD simulation was carried out for ZINC000000899161(Oxyresveratrol) using the most stable pose from IFD for 100 ns duration to detect the stability of protein–ligand complexes and interactions. By interpreting RMSD value the structural stability of protein–ligand was analyzed. The protein 4LXZ and ZINC000000899161 docked structure represent complex 1. During MD stimulation, for every 100 ps one frame was acquired, which further was saved generating 1000 frames throughout the stimulation.

**Figure 3:**
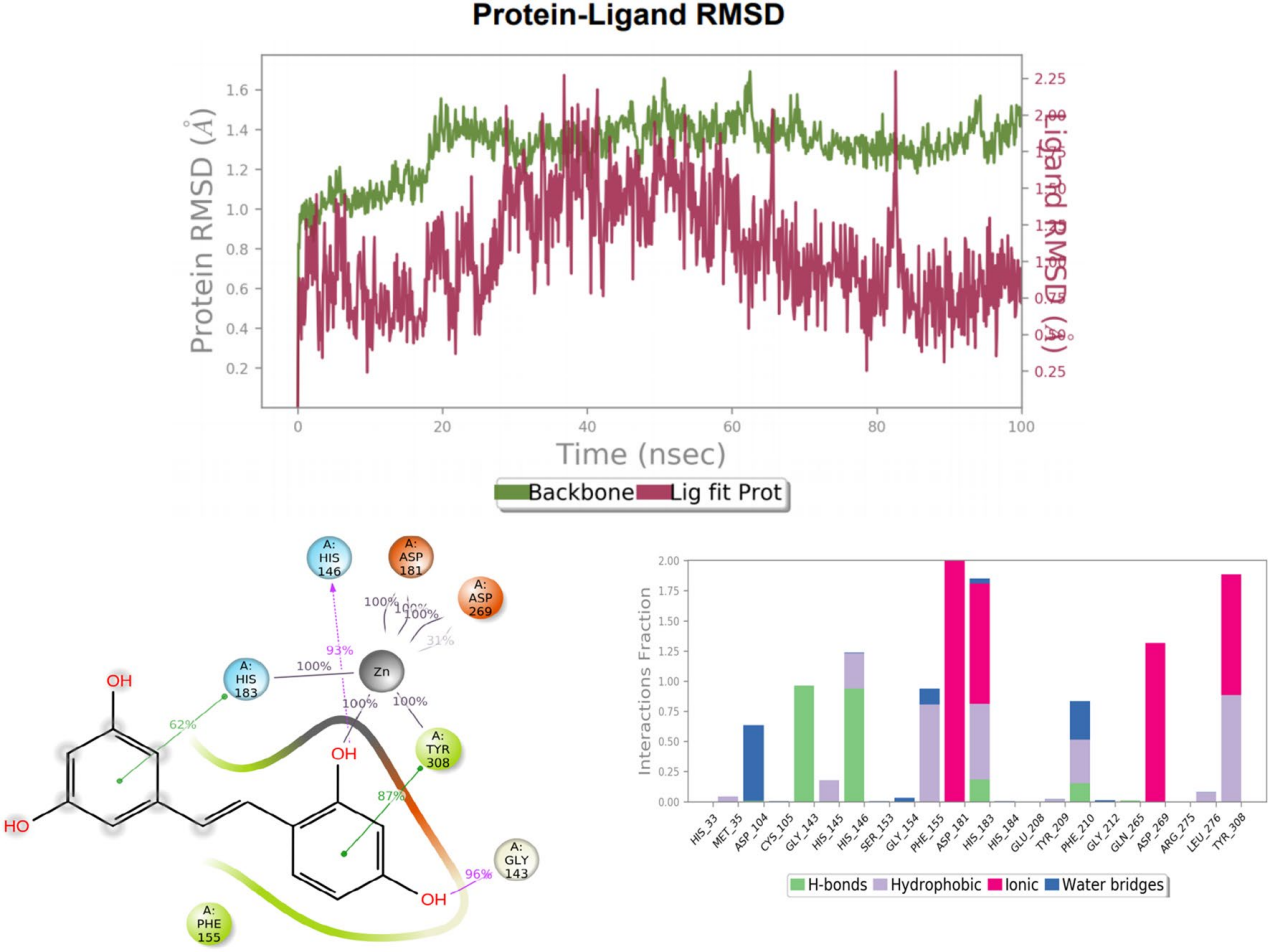
Image representing the protein ligand RMSD (ZINC000000899161), protein–ligand contacts and interacting amino acid residues observed during 100 ns MD simulation study.

In complex 1, RMSD value for 4LXZ protein was found to be 2 and ZINC000000899161 showed RMSD score 0.90. The complex 1 was initially stable for 0 to 5 ns, and 30–50 ns. The drift was observed for 4–30 ns and 50–100 ns. Further, the MD ligand–protein interactions were analyzed for both complexes, as compared to ligand–protein interactions. In complex 1, new pi-pi stacking interactions were found with TYR308, retained with HIS183, and missed with PHE210. Similarly, GLY143 formed new H-bond, H-bond was retained with HIS146 and lost with HIS183. When compared to XP docking, the hydrophobic bond interactions were lost with LEU276, PHE210, CYS156, MET35 and LEU144, and retained with TYR308 and PHE155. The polar interactions were retained with HIS146 and HIS183, while lost with other amino acid residues. The charged negative interactions were retained with ASP181 and lost with ASP269. The new charged negative interaction was found with ASP104. In complex 1, amino acid residues HIS183, TYR308, ASP181, ASP269 contributing significantly (100%) to metal coordination with ZN401. The MD ligand interaction diagrams of complex 1 are given in Fig. 3. After analyzing the data from MD studies, the molecule, ZINC000000899161(oxyresveratrol), was selected for further in-vivo studies. The interaction of the molecule with the protein at 100 ns were evaluated and it showed similar interactions as that of the In-bound ligand SAHA.

### Effect of ORV on escape latency and path efficiency in STZ-induced AD rats

**Figure 4:**
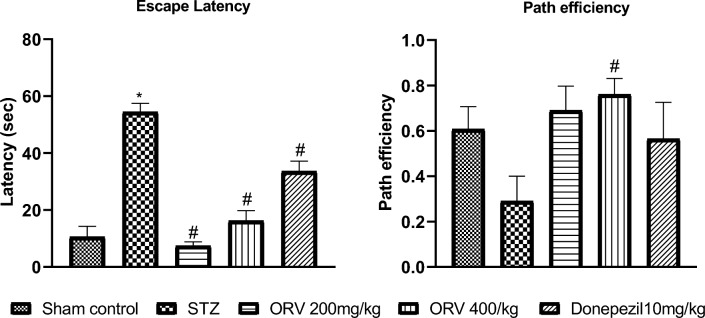
Effect of ORV on escape latency and path efficiency in STZ-induced AD rats. Data are expressed as Mean ± SEM. *p* < 0.05 was considered statistically significant. STZ, Streptozotocin; ORV, oxyresveratrol-betacyclodextrin. *p < 0.05: as compared to sham control. #*p* < 0.05: when compared to STZ group.

In the STZ-induced AD rats, the escape latency significantly increased in comparison to the sham control (*p* < 0.05). However, the ORV in STZ + ORV groups dose-dependently and significantly reduced the escape latency compared to the STZ-induced AD (*p* < 0.05). Similarly, the standard drug, donepezil, significantly reduced the latency period compared to the STZ-induced AD, although with lesser efficacy compared to the ORV doses. Path efficiency increased in all the groups in comparison to disease control group, however significant increase was seen in rats treated with higher dose of ORV. Figure 4 presents the effect of ORV on escape latency and path efficiency in experimental AD rats.

### Effect of ORV on acetylcholinesterase activity in STZ-induced AD rats

**Figure 5:**
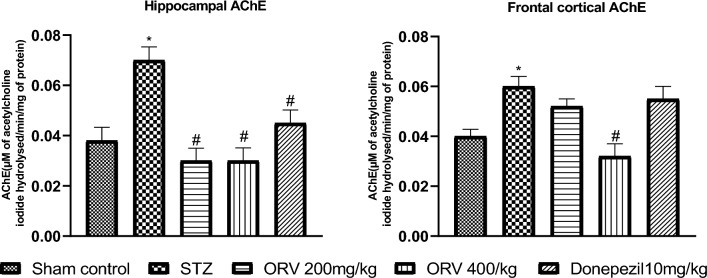
Effect of ORV on acetylcholinesterase activity in STZ-induced AD rats. Data are expressed as Mean ± SEM. *p* < 0.05 was considered statistically significant. STZ, Streptozotocin; ORV, oxyresveratrol-betacyclodextrin. *p < 0.05: significantly different when compared to sham group. #*p* < 0.05: significantly different when compared to STZ group.

In rats with STZ-induced Alzheimer's disease, there was a noticeable increase in AChE activity in the hippocampus and frontal cortex compared to the sham control group. On the contrary, the low and high doses of ORV as well as the standard drug appreciably reduced the activity of AChE in the hippocampus, whereas only the high dose significantly reduced the AChE level in the frontal cortex region of rats (*p* < 0.05). The effect of ORV on the hippocampal and cortical acetylcholinesterase (AChE) activity in STZ-induced AD rats has been depicted in Fig. 5.

### Effect of ORV on malondialdehyde level in STZ-induced AD rats

**Figure 6:**
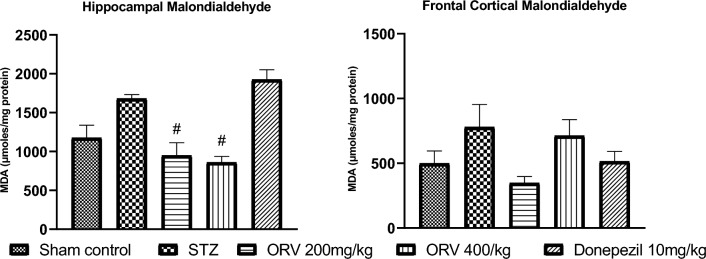
Effect of ORV on malondialdehyde in STZ-induced AD rats. Data are expressed as Mean ± SEM. *p* < 0.05 was considered statistically significant. STZ, Streptozotocin; ORV, oxyresveratrol-betacyclodextrin. *p < 0.05: significantly different when compared to sham group. #*p* < 0.05: significantly different when compared to STZ group.

The STZ-induced AD in rats increased the malondialdehyde (MDA) level in the frontal cortex and hippocampus compared to the sham control. On the contrary, the doses of ORV and standard drug appreciably reduced the hippocampal level of MDA, whereas only the low dose significantly reduced the frontal cortical level of MDA in the rats (*p* < 0.05). Figure 6 depicts the effect of ORV on the hippocampal and cortical MDA in STZ-induced AD rats.

### Effect of ORV on histone deacetylase activity in STZ-induced AD rats

**Figure 7:**
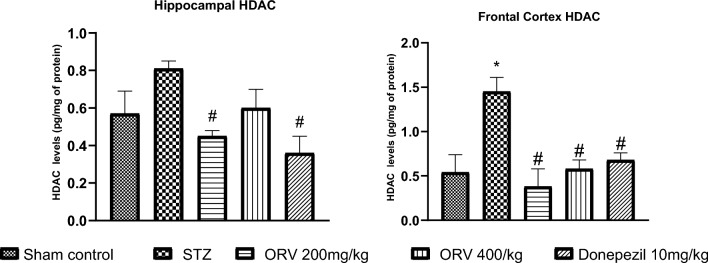
Effect of ORV on histone deacetylase activity in STZ-induced AD rats. Data are expressed as Mean ± SEM. *p* < 0.05 was considered statistically significant. STZ, Streptozotocin; ORV, oxyresveratrol-betacyclodextrin. *p < 0.05: significantly different when compared to sham group, #*p* < 0.05: significantly different when compared to STZ group.

The STZ-induced AD rats significantly increased the HDAC activity in the hippocampus and frontal cortex compared to the sham control (*p* < 0.05). On the contrary, the low dose of ORV and the standard drug remarkably reduced the hippocampal HDAC activity, whereas the ORV doses and the standard drug significantly reduced the activity of HDAC in the rats’ frontal cortex (*p* < 0.05) compared to STZ group. Figure 7 shows the effect of ORV on the hippocampal and cortical HDAC activity in STZ-induced AD rats.

### Histopathological examination

**Figure 8:**
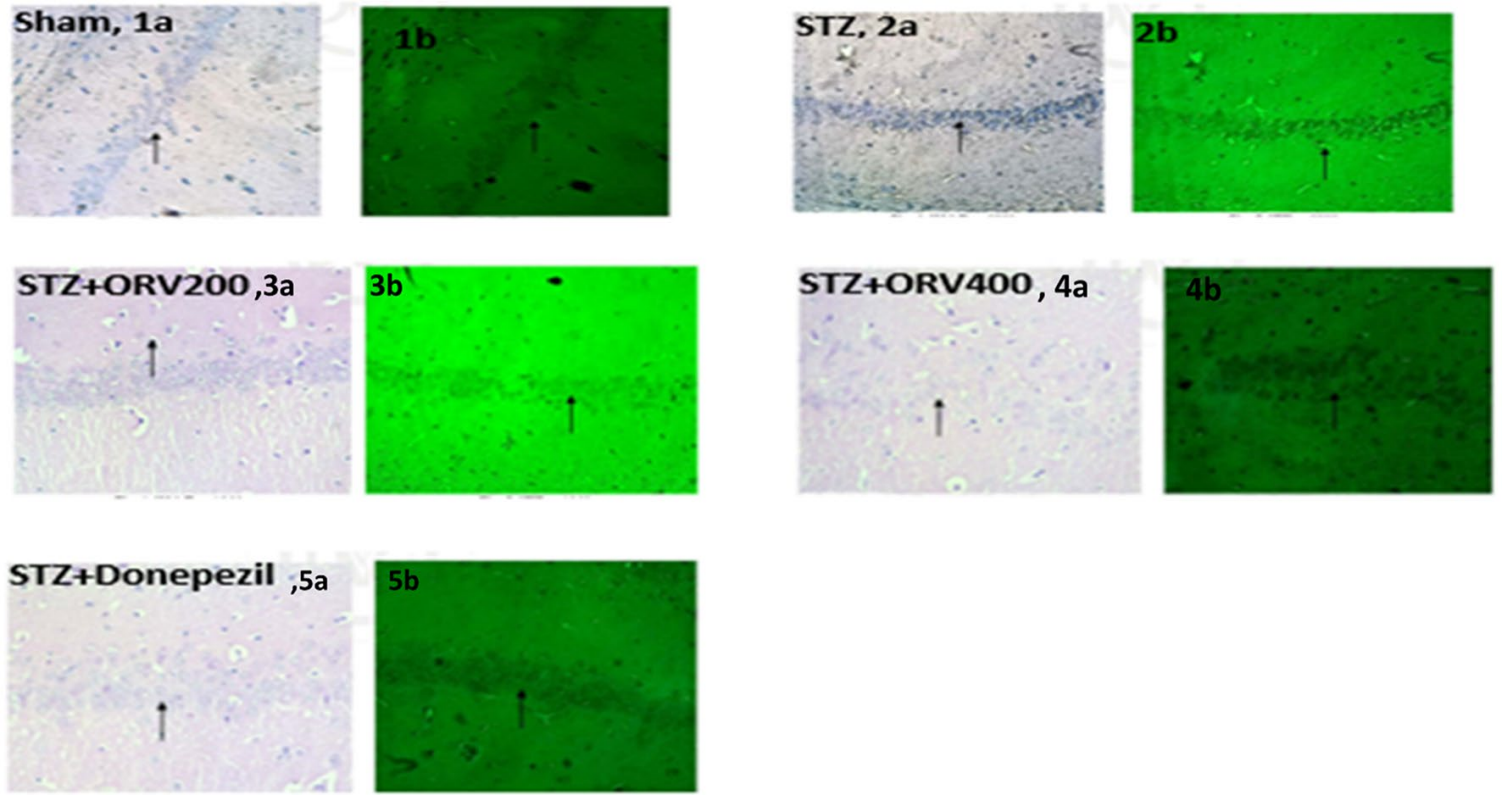
Effect of ORV doses (200 and 400 mg/kg) and donepezil standard drug on hippocampus of STZ-induced AD in rats. Normal sham control showed normal neurons structure without shrinkage (grey, 1a) and plaque (green, 1b). STZ group showed hyperchromatic nucleus, shrunken cells (grey, 2a), and neurofibrillary tangles (green, 2b). STZ + ORV200 and 400 groups revealed few shrunken cells (grey, 3a, 4a), sparse/no neurofibrillary tangles (green, 3b, 4b). STZ + Donepezil showed structures consistent with the treatment groups above.

**Figure 9:**
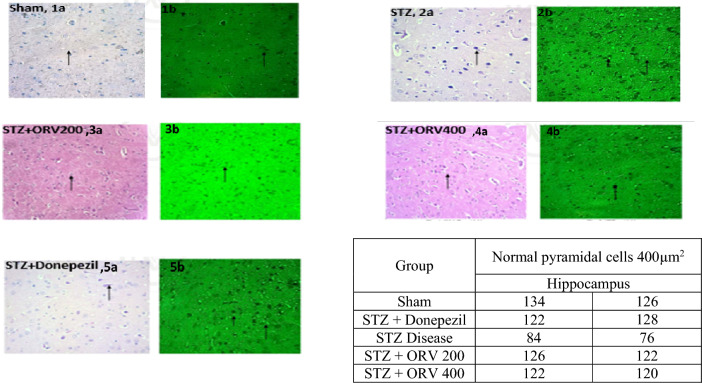
Effect of ORV doses (200 and 400 mg/kg) and donepezil standard drug on frontal cortex of STZ-induced AD in rats. Normal sham control showed normal pyramidal microglial cells (grey, 1a), with no plaques and tangles (green, 1b). STZ-AD group showed shrunken pyramidal cells (grey, 2a) with neurofibrillary tangles (2b). In the STZ + ORV group intact pyramidal cells and mild increase in microglial cells (grey, 3a, 4a), sparse/no neurofibrillary tangles (green, 3b, 4b). The STZ + Donepezil standard group revealed similar features as treatment group in the cortex and hippocampus.

The histopathological results from the brain regions in this study are presented in Figs. 8 and 9. Two stains (H & E and Thioflavin) were used to view alterations. The sham control group showed normal densely packed neurons having nucleus and granules in the cytoplasm without shrinkage (arrow, 1a); there were no neuritic plaques and neurofibrillary tangles (arrow, 1b). In the STZ-AD group, there were hyperchromatic nucleus with granules in the cytoplasm and shrunken cells (arrow, 2a), thioflavin stain showed neurofibrillary tangles (arrow, 2b). In the STZ + ORV200 group, there were pale nuclei and granules in the cytoplasm with few shrunken cells (arrow, 3a), sparse neurofibrillary tangles (arrow, 3b). In the STZ + ORV400 group, there were pale nuclei and granules in the cytoplasm without shrinkage (arrow, 4a), no neuritic plaques and neurofibrillary tangles (arrow, 4b). In the cortex of sham control, intact pyramidal microglial cells were observed (arrow, 1a), there were no neuritic plaques and neurofibrillary tangles (arrow, 1b). In the STZ-AD group, there were focal shrunken pyramidal cells with neurofibrillary tangles (arrow, 2a), thioflavin stain showed neurofibrillary tangles (arrow, 2b). In the STZ + ORV200 group, there were intact pyramidal cells and mild increase in microglial cells. The blood vessels appear intact (arrow, 3a), sparse neurofibrillary tangles (arrow, 3b). In the STZ + ORV400 group, intact pyramidal cells and mild increase in microglial cells (arrow, 4a), no neuritic plaques and neurofibrillary tangles (arrow, 4b). The STZ + Donepezil standard group revealed similar features as treatment group in the cortex and hippocampus.

## Discussion

The current study describes the evaluation of the neuroprotective activity of ORV and deciphering the involvement of HDAC mediated epigenetic modulation in its activity using in silico and in vivo method. Computational study done by Pham-the et al. showed that the dock score of in-bound ligand SAHA to be − 8.995^35^ and demonstrated that SAHA formed hydrogen bond interactions with, ASP104, TYR308, HIS145, HIS146, and ASP 181, as well as hydrophobic interaction with PHE155 and PHE210. Taking this into considerations, the phytochemical database was docked on the active motif of HDAC 2. All 10 Drugs showed similar interactions as compared to SAHA. Metal coordination with Zn401 and hydrogen bond with TYR308 were the common interaction between the selected 10 molecules and SAHA. Zn interaction is important as HDAC inhibitor consist of zinc-binding motif. All the selected molecules were interacting with at least 3 amino acid residue as mentioned above for SAHA^36^. The vital interaction such as hydrogen bond with TYR308 and metal coordination with Zn can possibly be responsible for HDAC inhibition. Also, few hydrogen bond interactions were seen with GLU103, GLU208, TYR209. Amino acid residue such as ASP269, LEU276, ASP181, PRO34 were the non-bonding interaction near the active motif of HDAC inhibitor.

Streptozotocin (STZ) is a glucosamine-nitrosourea used to induce experimental diabetes in animals due to its targeted toxic effect on pancreatic β-cells. However, convincing evidence has shown that STZ injection via intracebroventricular (ICV) or intraperitoneal route produces sporadic AD pathology in experimental animals^3,37,38^. STZ triggers cognition deficits, increased cerebral aggregated Aβ fragments, tau protein and Aβ deposits which are hallmarks of sporadic AD in humans^3,37^. In this study, STZ, via ICV injection, induced AD-like sporadic dementia with histologic neuronal lesions and neurofibrillary tangles^31,39^. The behavioural study revealed that the escape latency period significantly increased in the STZ-AD rats compared to the control (Fig. 3). Escape latency is a parameter for spatial memory measurement in animal models^40^. It is known that an increase in the time of rat’s escape from the maze indicates decreased in spatial and emotional memory and lesions in the hippocampus^40,41^. Therefore, the increased escape latency in our result depicts lowered memory in the rats in consonance with the earlier reports^39,41^. Conversely, ORV and donepezil markedly reduced the escape latency in the STZ-AD rats. This reveals the potential of ORV to improve memory in AD pathology. However, although oxyresveratrol has demonstrated beneficial health effect against experimental AD in previous studies^22,42^, this is the first study to report the possible beneficial health effect of ORV against AD rats. Additionally, it is noteworthy that ORV at 200 mg/kg body weight appeared to have highest reducing effect on the escape latency period of rats in this study.

Neurological deficit is strongly associated with altered AChE activity in the brain. Increased activities of AChE and butyrylcholinesterase and the corresponding decreases in acetylcholine and butyrylcholine levels, respectively, are characteristic features of AD^2,43^. The FDA-approved AChE inhibitors, including donepezil, modulate levels of acetylcholine, an important neurotransmitter that is responsible for memory and cognitive function in the brain^44^. Thus, argumentation of cholinergic transmission has been suggested as an important therapeutic target in AD pathophysiology^1^. Given the above existing reports, our study explored the effect of STZ-AD and ORV on AChE activity in the frontal cortex and hippocampus of rats. The induction of the experimental AD in STZ-AD rats caused considerable increases in the cortical and hippocampal activities of AChE compared to the control (Fig. 4). By implication, the increased AChE activity would trigger elevated hydrolysis of acetylcholine to choline and acetate implicated in AD symptoms^1^. Interestingly, the induction of oxidative stress in the brain regions is indicated in this study by marked increase in MDA level. It is well known that oxidative stress and/or ROS generation is a key player in cognition deficit and AD pathogenesis, and may underlie the increased AChE activity in this study^3,45^. MDA is a peroxidative marker of oxidative stress, and it might have emanated from the altered oxidative balance in this study. Herein, MDA levels increased significantly in the frontal cortex and hippocampus of the AD rats (Fig. 5). This observation indicates oxidative imbalance which has been earlier reported to incite elevated activity of AChE^46^. It could be conceived in the current study that the elevated AChE activities in the brain regions disrupt the cholinergic transmission via oxidative stress which is a priority in the severity of AD dementia. Our finding is in agreement with the studies of Falode et al. (2017) and Elmorsy et al. (2021). On the other hand, the administration of ORV reversed the AChE activity in the brain regions as well as the MDA levels in this study. Interestingly, ORV and donepezil prominently decreased the AChE activity and MDA levels compared to the STZ-AD group. However, the opposite effects of ORV were observed to be more pronounced than the standard drug. Moreover, there was a dose-dependent depression of AChE by the two doses of ORV in this study. Taken together therefore, the excelling depression of AChE and mitigation of oxidative stress by ORV could be due to its solubility or in part its enhanced bioavailability de novo compared to the standard drug, the AChE inhibitor (Supplementary information).

In neurodegenerative diseases such as AD, robust evidence indicates that histone deacetylation is one of the key culprits that mediate AD progression. The histone acetylation is compromised in AD, while hyperacetylation promotes neuroprotective actions^7^. In this study, the induction of AD increased the activities of HDAC in the cortical and hippocampal regions of the rats’ brain following previous studies (Fig. 6)^39,47^. In addition, the neuronal lesions of hyperchromatic nucleus, shrunken cells, and neurofibrillary tangles were observed in AD rat histology (Figs. 7, 8). The mechanism underlying the accumulating findings is currently unclear; however, impairment in HDAC homeostasis linked with amyloid-β (Aβ) deposition and cognitive dysfunction have been highlighted in the literature^47^. The elevated expression of HDAC in this study confirms the cognitive dysfunction herein suggested by our escape latency results. Akone et al.^18^ chronicles the natural products that inhibit HDAC expression, and oxyresveratrol has been shown to prevent key hallmarks of AD pathology^22,42^, we hereby report that ORV considerably reduced the HDAC activity in the frontal cortex and hippocampus in this study. However, we observed that the donepezil was more effective in the hippocampus, whereas the 200 mg/kg dose of ORV was more effective in the cortex. This may be due, in part, to nature or responses of different cells in the brain regions to the agents. At the same time, it suggests that the optimal therapeutic effect of ORV is around 200 mg/kg, and this may be importantly considered in future studies. The anti-HDAC behaviour of ORV could be attributed possibly to its antioxidant, anti-inflammatory and strong neuroprotective activities already reported in the published papers^22,25^.

## Conclusion

In conclusion, intracebroventricular STZ injection could produce AD-like dementia model used in the current study. For the first time, our study presents findings on the potential of ORV in preventing cognitive dysfunction via mitigation of oxidative stress and inhibition of HDAC protein expression in the frontal cortex and hippocampus of STZ-induced AD in rats. This study suggests that the lower dose of ORV (200 mg/kg) may exert appealing effects than the higher dose, suggesting that the optimal therapeutic effect of ORV is around 200 mg/kg. Nevertheless, future studies may unravel the mechanisms underlying the protective effect of ORV.

### Supplementary Information

Supplementary Information.

## Data Availability

The authors confirm that the data supporting the findings of this study are available within the article.
