# Supplementary material for: Oxyresveratrol-β-cyclodextrin mitigates streptozotocin-induced Alzheimer's model cognitive impairment, histone deacetylase activity in rats: in silico & in vivo studies
Source: Sci Rep. 2024 Apr 30;14:9897. doi: 10.1038/s41598-024-57188-7 (PMC11061296; doi:10.1038/s41598-024-57188-7)

Supplementary Fig 1. Figure showing the difference between group (Mean) for escape latency.

Supplementary Table 1: Detailed statistical parameters showing comparison between groups for escape latency.

| Test details | Mean 1 | Mean 2 | Mean Diff. | SE of diff. | n1 | n2 | q | DF |
| --- | --- | --- | --- | --- | --- | --- | --- | --- |
| Sham control vs. STZ | 10.60 | 54.44 | -43.84 | 4.488 | 8 | 8 | 13.81 | 35 |
| Sham control vs. ORV 200mg/kg | 10.60 | 7.420 | 3.180 | 4.488 | 8 | 8 | 1.002 | 35 |
| Sham control vs. ORV 400/kg | 10.60 | 16.20 | -5.600 | 4.488 | 8 | 8 | 1.765 | 35 |
| Sham control vs. Donepezil 10mg/kg | 10.60 | 33.60 | -23.00 | 4.488 | 8 | 8 | 7.248 | 35 |
| STZ vs. ORV 200mg/kg | 54.44 | 7.420 | 47.02 | 4.488 | 8 | 8 | 14.82 | 35 |
| STZ vs. ORV 400/kg | 54.44 | 16.20 | 38.24 | 4.488 | 8 | 8 | 12.05 | 35 |
| STZ vs. Donepezil 10mg/kg | 54.44 | 33.60 | 20.84 | 4.488 | 8 | 8 | 6.567 | 35 |
| ORV 200mg/kg vs. ORV 400/kg | 7.420 | 16.20 | -8.780 | 4.488 | 8 | 8 | 2.767 | 35 |
| ORV 200mg/kg vs. Donepezil 10mg/kg | 7.420 | 33.60 | -26.18 | 4.488 | 8 | 8 | 8.250 | 35 |
| ORV 400/kg vs. Donepezil 10mg/kg | 16.20 | 33.60 | -17.40 | 4.488 | 8 | 8 | 5.483 | 35 |

Supplementary Fig 2. Figure showing the difference between group (Mean) for path efficiency.

Supplementary Table 2: Detailed statistical parameters showing comparison between groups for path efficiency.

| Test details | Mean 1 | Mean 2 | Mean Diff. | SE of diff. | n1 | n2 | q | DF |
| --- | --- | --- | --- | --- | --- | --- | --- | --- |
| Sham control vs. STZ | 0.6070 | 0.2900 | 0.3170 | 0.1605 | 8 | 8 | 2.794 | 35 |
| Sham control vs. ORV 200mg/kg | 0.6070 | 0.6900 | -0.08300 | 0.1605 | 8 | 8 | 0.7315 | 35 |
| Sham control vs. ORV 400/kg | 0.6070 | 0.7610 | -0.1540 | 0.1605 | 8 | 8 | 1.357 | 35 |
| Sham control vs. Donepezil 10mg/kg | 0.6070 | 0.5650 | 0.04200 | 0.1605 | 8 | 8 | 0.3702 | 35 |
| STZ vs. ORV 200mg/kg | 0.2900 | 0.6900 | -0.4000 | 0.1605 | 8 | 8 | 3.525 | 35 |
| STZ vs. ORV 400/kg | 0.2900 | 0.7610 | -0.4710 | 0.1605 | 8 | 8 | 4.151 | 35 |
| STZ vs. Donepezil 10mg/kg | 0.2900 | 0.5650 | -0.2750 | 0.1605 | 8 | 8 | 2.424 | 35 |
| ORV 200mg/kg vs. ORV 400/kg | 0.6900 | 0.7610 | -0.07100 | 0.1605 | 8 | 8 | 0.6258 | 35 |
| ORV 200mg/kg vs. Donepezil 10mg/kg | 0.6900 | 0.5650 | 0.1250 | 0.1605 | 8 | 8 | 1.102 | 35 |
| ORV 400/kg vs. Donepezil 10mg/kg | 0.7610 | 0.5650 | 0.1960 | 0.1605 | 8 | 8 | 1.727 | 35 |

Supplementary Fig 3. Figure showing the difference between group (Mean) for hippocampal AChE.

Supplementary Table 3: Detailed statistical parameters showing comparison between groups for hippocampal AChE.

| Test details | Mean 1 | Mean 2 | Mean Diff. | SE of diff. | n1 | n2 | q | DF |
| --- | --- | --- | --- | --- | --- | --- | --- | --- |
| Sham control vs. STZ | 0.03800 | 0.07000 | -0.03200 | 0.007327 | 8 | 8 | 6.176 | 35 |
| Sham control vs. ORV 200mg/kg | 0.03800 | 0.03000 | 0.008000 | 0.007327 | 8 | 8 | 1.544 | 35 |
| Sham control vs. ORV 400/kg | 0.03800 | 0.03000 | 0.008000 | 0.007327 | 8 | 8 | 1.544 | 35 |
| Sham control vs. Donepezil 10mg/kg | 0.03800 | 0.04500 | -0.007000 | 0.007327 | 8 | 8 | 1.351 | 35 |
| STZ vs. ORV 200mg/kg | 0.07000 | 0.03000 | 0.04000 | 0.007327 | 8 | 8 | 7.720 | 35 |
| STZ vs. ORV 400/kg | 0.07000 | 0.03000 | 0.04000 | 0.007327 | 8 | 8 | 7.720 | 35 |
| STZ vs. Donepezil 10mg/kg | 0.07000 | 0.04500 | 0.02500 | 0.007327 | 8 | 8 | 4.825 | 35 |
| ORV 200mg/kg vs. ORV 400/kg | 0.03000 | 0.03000 | 0.000 | 0.007327 | 8 | 8 | 0.000 | 35 |
| ORV 200mg/kg vs. Donepezil 10mg/kg | 0.03000 | 0.04500 | -0.01500 | 0.007327 | 8 | 8 | 2.895 | 35 |
| ORV 400/kg vs. Donepezil 10mg/kg | 0.03000 | 0.04500 | -0.01500 | 0.007327 | 8 | 8 | 2.895 | 35 |

Supplementary Fig 4. Figure showing the difference between group (Mean) for cortical AChE.

Supplementary Table 4: Detailed statistical parameters showing comparison between groups for cortical AChE.

| Test details | Mean 1 | Mean 2 | Mean Diff. | SE of diff. | n1 | n2 | q | DF |
| --- | --- | --- | --- | --- | --- | --- | --- | --- |
| Sham control vs. STZ | 0.04000 | 0.06000 | -0.02000 | 0.005756 | 8 | 8 | 4.914 | 35 |
| Sham control vs. ORV 200mg/kg | 0.04000 | 0.05200 | -0.01200 | 0.005756 | 8 | 8 | 2.948 | 35 |
| Sham control vs. ORV 400/kg | 0.04000 | 0.03200 | 0.008000 | 0.005756 | 8 | 8 | 1.965 | 35 |
| Sham control vs. Donepezil10mg/kg | 0.04000 | 0.05500 | -0.01500 | 0.005756 | 8 | 8 | 3.685 | 35 |
| STZ vs. ORV 200mg/kg | 0.06000 | 0.05200 | 0.008000 | 0.005756 | 8 | 8 | 1.965 | 35 |
| STZ vs. ORV 400/kg | 0.06000 | 0.03200 | 0.02800 | 0.005756 | 8 | 8 | 6.879 | 35 |
| STZ vs. Donepezil10mg/kg | 0.06000 | 0.05500 | 0.005000 | 0.005756 | 8 | 8 | 1.228 | 35 |
| ORV 200mg/kg vs. ORV 400/kg | 0.05200 | 0.03200 | 0.02000 | 0.005756 | 8 | 8 | 4.914 | 35 |
| ORV 200mg/kg vs. Donepezil10mg/kg | 0.05200 | 0.05500 | -0.003000 | 0.005756 | 8 | 8 | 0.7370 | 35 |
| ORV 400/kg vs. Donepezil10mg/kg | 0.03200 | 0.05500 | -0.02300 | 0.005756 | 8 | 8 | 5.651 | 35 |

Supplementary Fig 5. Figure showing the difference between group (Mean) for hippocampal MDA.

Supplementary Table 5: Detailed statistical parameters showing comparison between groups for hippocampal MDA.

| Test details | Mean 1 | Mean 2 | Mean Diff. | SE of diff. | n1 | n2 | q | DF |
| --- | --- | --- | --- | --- | --- | --- | --- | --- |
| Sham control vs. STZ | 1175 | 1680 | -505.2 | 177.9 | 8 | 8 | 4.016 | 35 |
| Sham control vs. ORV 200mg/kg | 1175 | 948.3 | 226.7 | 177.9 | 8 | 8 | 1.802 | 35 |
| Sham control vs. ORV 400/kg | 1175 | 858.8 | 316.2 | 177.9 | 8 | 8 | 2.514 | 35 |
| Sham control vs. Donepezil 10mg/kg | 1175 | 1925 | -750.3 | 177.9 | 8 | 8 | 5.964 | 35 |
| STZ vs. ORV 200mg/kg | 1680 | 948.3 | 731.9 | 177.9 | 8 | 8 | 5.818 | 35 |
| STZ vs. ORV 400/kg | 1680 | 858.8 | 821.4 | 177.9 | 8 | 8 | 6.530 | 35 |
| STZ vs. Donepezil 10mg/kg | 1680 | 1925 | -245.1 | 177.9 | 8 | 8 | 1.948 | 35 |
| ORV 200mg/kg vs. ORV 400/kg | 948.3 | 858.8 | 89.50 | 177.9 | 8 | 8 | 0.7115 | 35 |
| ORV 200mg/kg vs. Donepezil 10mg/kg | 948.3 | 1925 | -977.0 | 177.9 | 8 | 8 | 7.766 | 35 |
| ORV 400/kg vs. Donepezil 10mg/kg | 858.8 | 1925 | -1067 | 177.9 | 8 | 8 | 8.478 | 35 |

Supplementary Fig 6. Figure showing the difference between group (Mean) for cortical MDA.

Supplementary Table 6: Detailed statistical parameters showing comparison between groups for cortical MDA.

| Test details | Mean 1 | Mean 2 | Mean Diff. | SE of diff. | n1 | n2 | q | DF |
| --- | --- | --- | --- | --- | --- | --- | --- | --- |
| Sham control vs. STZ | 498.3 | 779.1 | -280.8 | 160.1 | 8 | 8 | 2.481 | 35 |
| Sham control vs. ORV 200mg/kg | 498.3 | 346.8 | 151.5 | 160.1 | 8 | 8 | 1.339 | 35 |
| Sham control vs. ORV 400/kg | 498.3 | 712.2 | -213.9 | 160.1 | 8 | 8 | 1.890 | 35 |
| Sham control vs. Donepezil 10mg/kg | 498.3 | 513.7 | -15.43 | 160.1 | 8 | 8 | 0.1363 | 35 |
| STZ vs. ORV 200mg/kg | 779.1 | 346.8 | 432.3 | 160.1 | 8 | 8 | 3.820 | 35 |
| STZ vs. ORV 400/kg | 779.1 | 712.2 | 66.88 | 160.1 | 8 | 8 | 0.5909 | 35 |
| STZ vs. Donepezil 10mg/kg | 779.1 | 513.7 | 265.3 | 160.1 | 8 | 8 | 2.344 | 35 |
| ORV 200mg/kg vs. ORV 400/kg | 346.8 | 712.2 | -365.4 | 160.1 | 8 | 8 | 3.229 | 35 |
| ORV 200mg/kg vs. Donepezil 10mg/kg | 346.8 | 513.7 | -166.9 | 160.1 | 8 | 8 | 1.475 | 35 |
| ORV 400/kg vs. Donepezil 10mg/kg | 712.2 | 513.7 | 198.5 | 160.1 | 8 | 8 | 1.754 | 35 |

Supplementary Fig 7. Figure showing the difference between group (Mean) for hippocampal HDAC level.

Supplementary Table 7: Detailed statistical parameters showing comparison between groups for hippocampal HDAC level.

| Test details | Mean 1 | Mean 2 | Mean Diff. | SE of diff. | n1 | n2 | q | DF |
| --- | --- | --- | --- | --- | --- | --- | --- | --- |
| Sham control vs. STZ | 0.5700 | 0.8100 | -0.2400 | 0.1225 | 8 | 8 | 2.771 | 42 |
| Sham control vs. ORV 200mg/kg | 0.5700 | 0.4500 | 0.1200 | 0.1225 | 8 | 8 | 1.386 | 42 |
| Sham control vs. ORV 400/kg | 0.5700 | 0.6000 | -0.03000 | 0.1225 | 8 | 8 | 0.3464 | 42 |
| Sham control vs. Donepezil 10mg/kg | 0.5700 | 0.3600 | 0.2100 | 0.1225 | 8 | 8 | 2.425 | 42 |
| Sham control vs. SAHA | 0.5700 | 0.3300 | 0.2400 | 0.1225 | 8 | 8 | 2.771 | 42 |
| STZ vs. ORV 200mg/kg | 0.8100 | 0.4500 | 0.3600 | 0.1225 | 8 | 8 | 4.157 | 42 |
| STZ vs. ORV 400/kg | 0.8100 | 0.6000 | 0.2100 | 0.1225 | 8 | 8 | 2.425 | 42 |
| STZ vs. Donepezil 10mg/kg | 0.8100 | 0.3600 | 0.4500 | 0.1225 | 8 | 8 | 5.196 | 42 |
| STZ vs. SAHA | 0.8100 | 0.3300 | 0.4800 | 0.1225 | 8 | 8 | 5.543 | 42 |
| ORV 200mg/kg vs. ORV 400/kg | 0.4500 | 0.6000 | -0.1500 | 0.1225 | 8 | 8 | 1.732 | 42 |
| ORV 200mg/kg vs. Donepezil 10mg/kg | 0.4500 | 0.3600 | 0.09000 | 0.1225 | 8 | 8 | 1.039 | 42 |
| ORV 200mg/kg vs. SAHA | 0.4500 | 0.3300 | 0.1200 | 0.1225 | 8 | 8 | 1.386 | 42 |
| ORV 400/kg vs. Donepezil 10mg/kg | 0.6000 | 0.3600 | 0.2400 | 0.1225 | 8 | 8 | 2.771 | 42 |
| ORV 400/kg vs. SAHA | 0.6000 | 0.3300 | 0.2700 | 0.1225 | 8 | 8 | 3.118 | 42 |
| Donepezil 10mg/kg vs. SAHA | 0.3600 | 0.3300 | 0.03000 | 0.1225 | 8 | 8 | 0.3464 | 42 |

Supplementary Fig 8. Figure showing the difference between group (Mean) for cortical HDAC level.

Supplementary Table 8: Detailed statistical parameters showing comparison between groups for cortical HDAC level.

| Test details | Mean 1 | Mean 2 | Mean Diff. | SE of diff. | n1 | n2 | q | DF |
| --- | --- | --- | --- | --- | --- | --- | --- | --- |
| Sham control vs. STZ | 0.5400 | 1.450 | -0.9100 | 0.2037 | 8 | 8 | 6.317 | 42 |
| Sham control vs. ORV 200mg/kg | 0.5400 | 0.3800 | 0.1600 | 0.2037 | 8 | 8 | 1.111 | 42 |
| Sham control vs. ORV 400/kg | 0.5400 | 0.5800 | -0.04000 | 0.2037 | 8 | 8 | 0.2777 | 42 |
| Sham control vs. Donepezil 10mg/kg | 0.5400 | 0.6800 | -0.1400 | 0.2037 | 8 | 8 | 0.9719 | 42 |
| Sham control vs. SAHA | 0.5400 | 0.4400 | 0.1000 | 0.2037 | 8 | 8 | 0.6942 | 42 |
| STZ vs. ORV 200mg/kg | 1.450 | 0.3800 | 1.070 | 0.2037 | 8 | 8 | 7.428 | 42 |
| STZ vs. ORV 400/kg | 1.450 | 0.5800 | 0.8700 | 0.2037 | 8 | 8 | 6.040 | 42 |
| STZ vs. Donepezil 10mg/kg | 1.450 | 0.6800 | 0.7700 | 0.2037 | 8 | 8 | 5.345 | 42 |
| STZ vs. SAHA | 1.450 | 0.4400 | 1.010 | 0.2037 | 8 | 8 | 7.012 | 42 |
| ORV 200mg/kg vs. ORV 400/kg | 0.3800 | 0.5800 | -0.2000 | 0.2037 | 8 | 8 | 1.388 | 42 |
| ORV 200mg/kg vs. Donepezil 10mg/kg | 0.3800 | 0.6800 | -0.3000 | 0.2037 | 8 | 8 | 2.083 | 42 |
| ORV 200mg/kg vs. SAHA | 0.3800 | 0.4400 | -0.06000 | 0.2037 | 8 | 8 | 0.4165 | 42 |
| ORV 400/kg vs. Donepezil 10mg/kg | 0.5800 | 0.6800 | -0.1000 | 0.2037 | 8 | 8 | 0.6942 | 42 |
| ORV 400/kg vs. SAHA | 0.5800 | 0.4400 | 0.1400 | 0.2037 | 8 | 8 | 0.9719 | 42 |
| Donepezil 10mg/kg vs. SAHA | 0.6800 | 0.4400 | 0.2400 | 0.2037 | 8 | 8 | 1.666 | 42 |


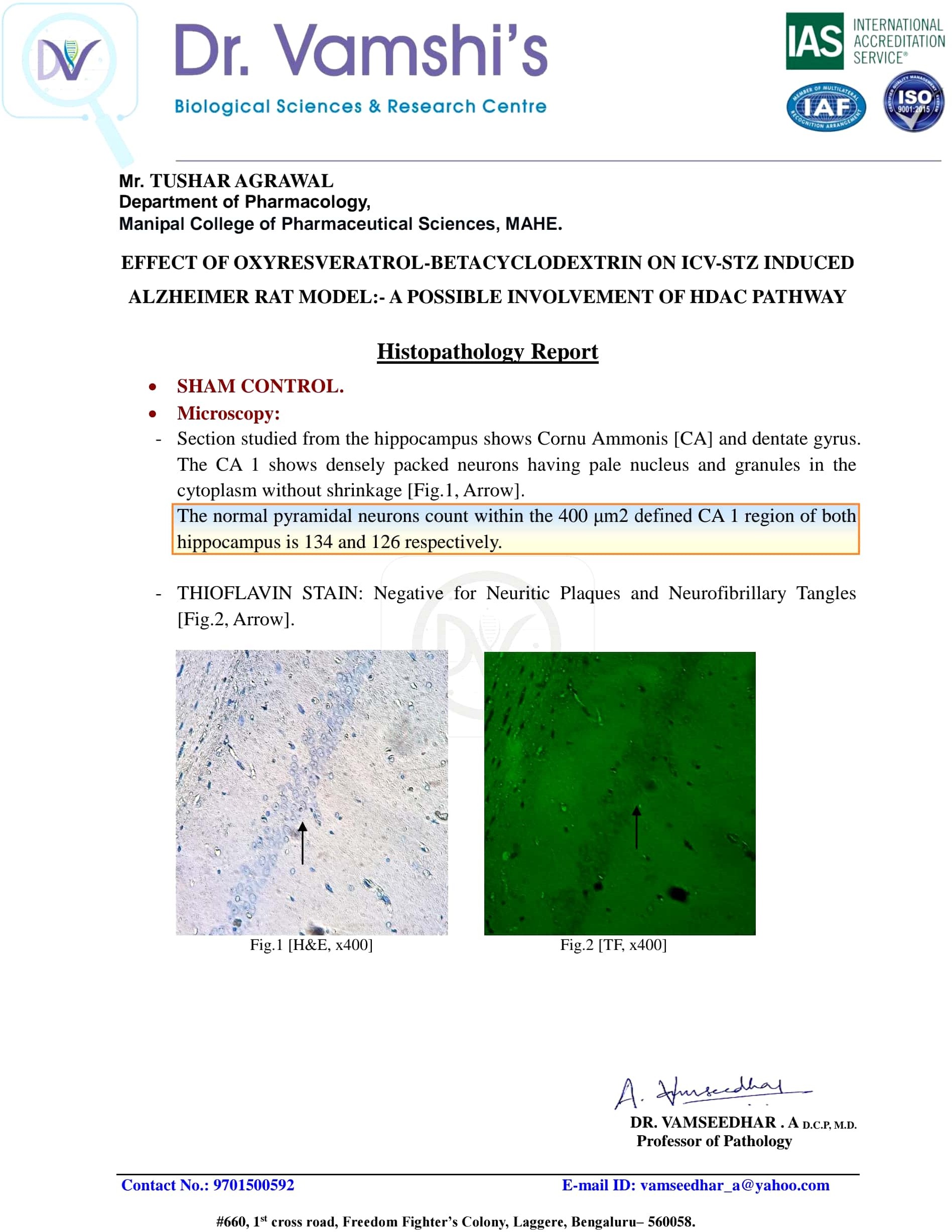


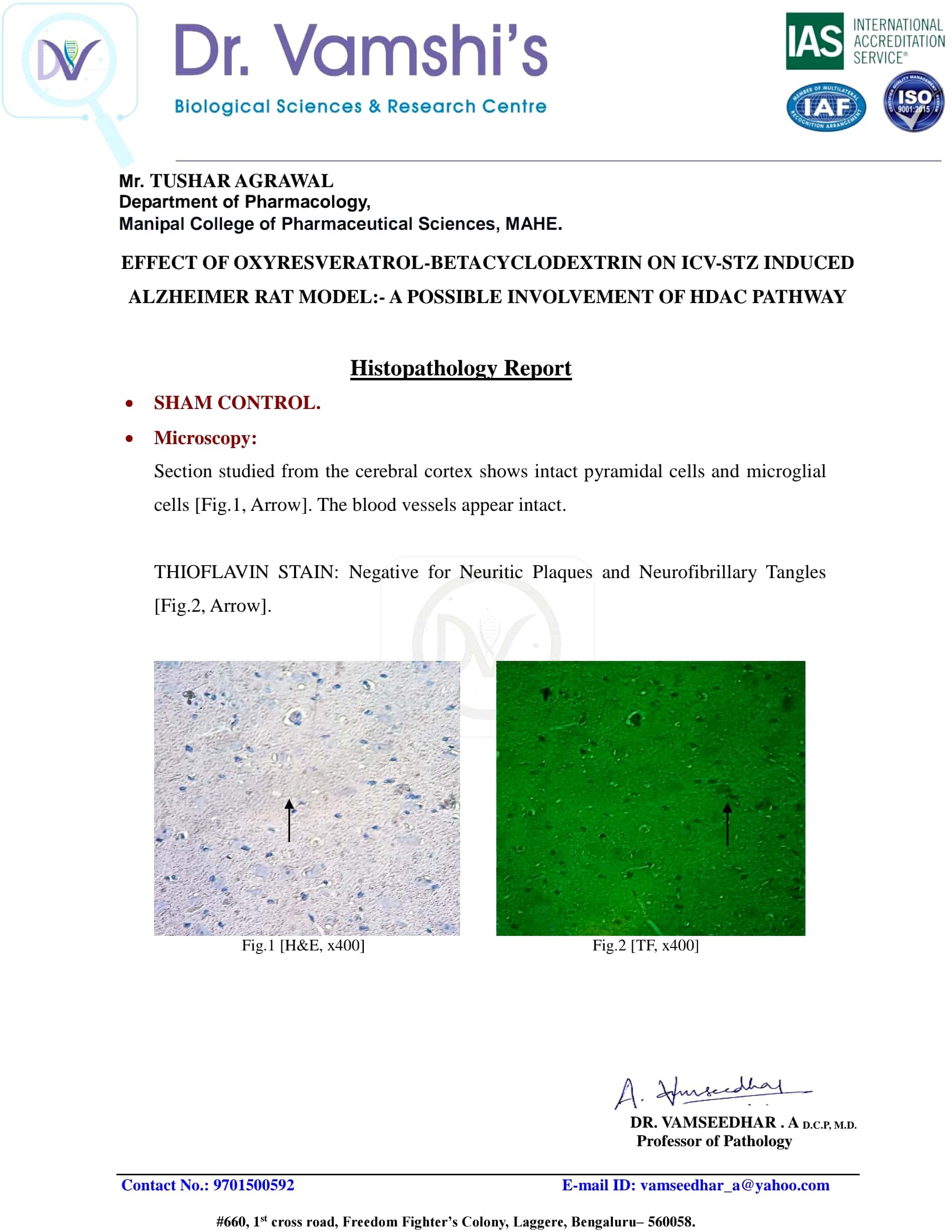


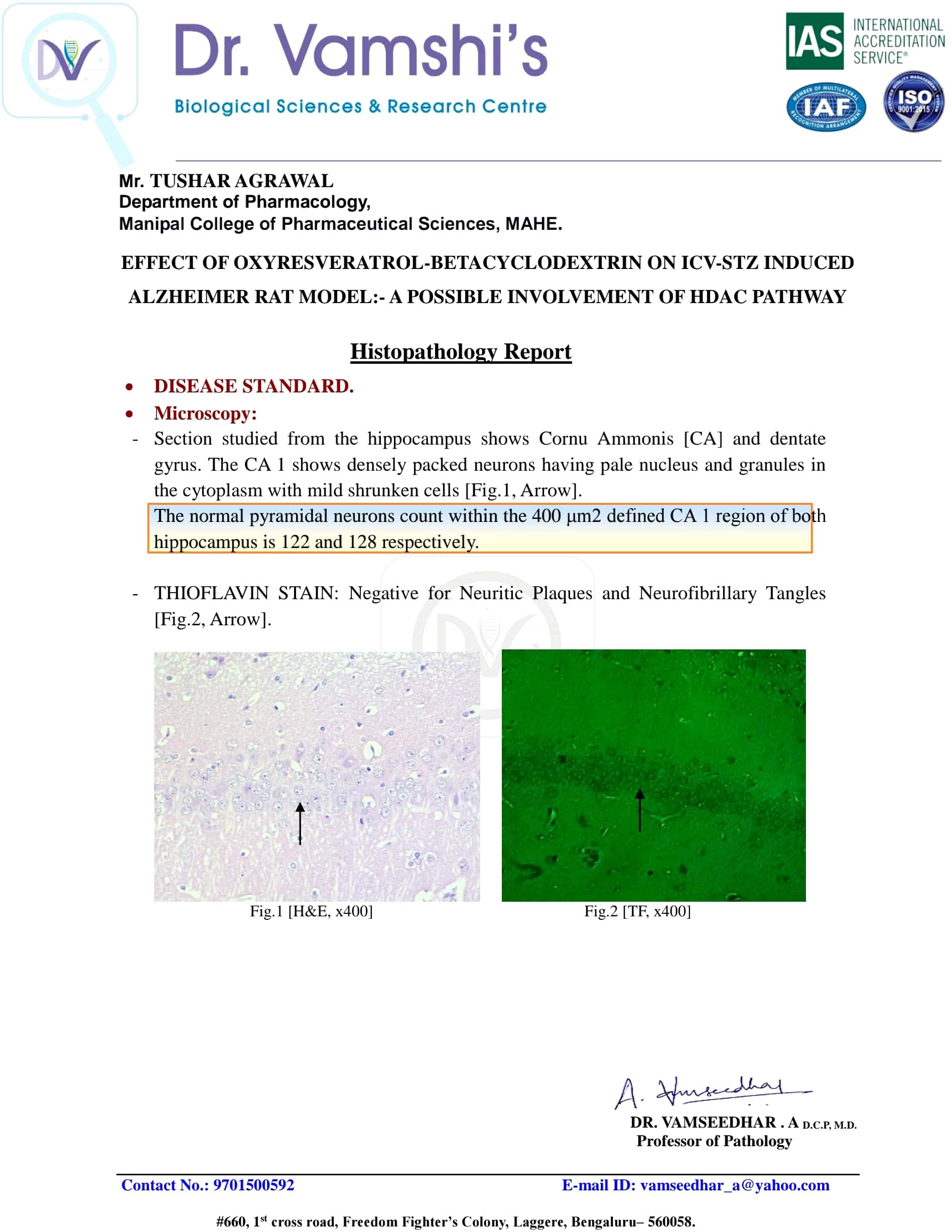


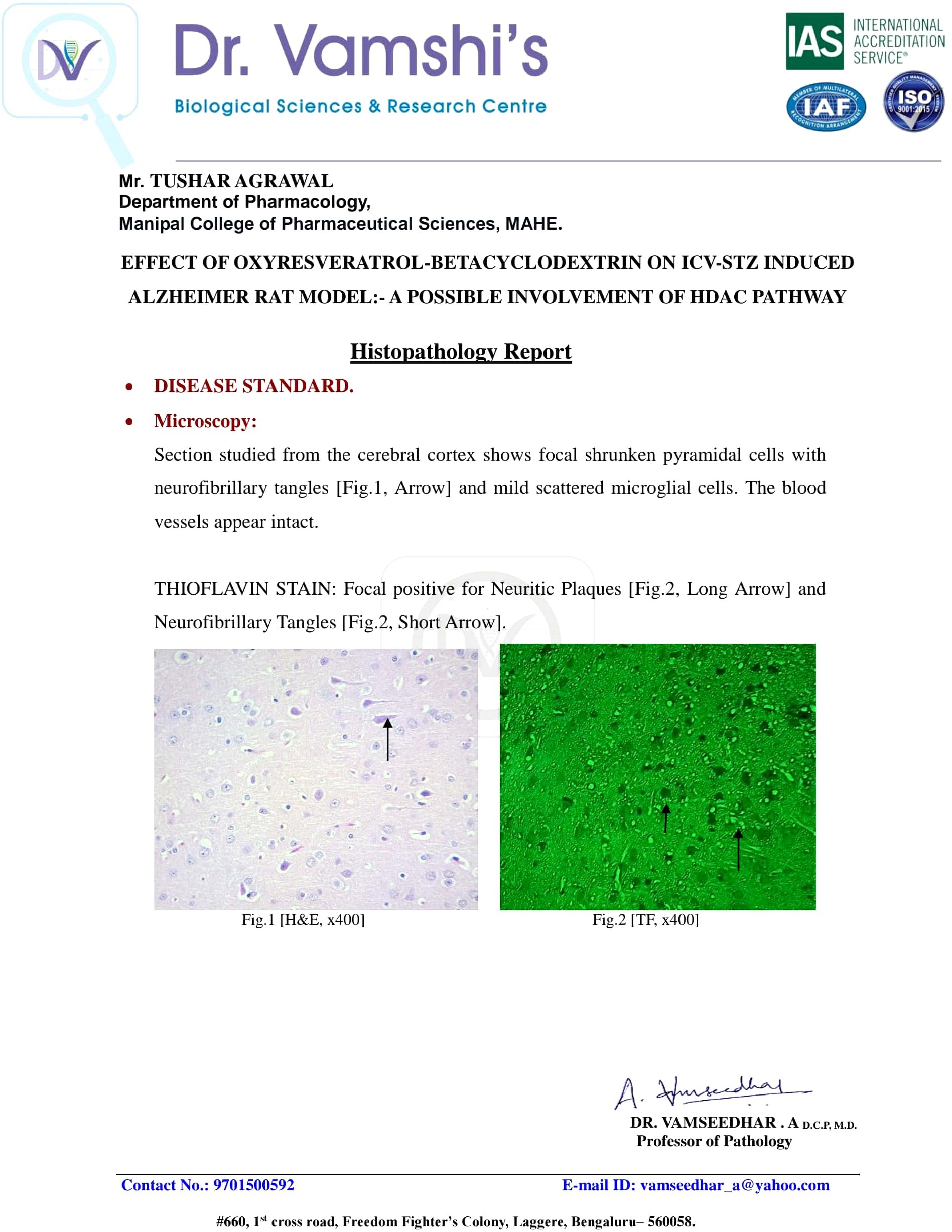


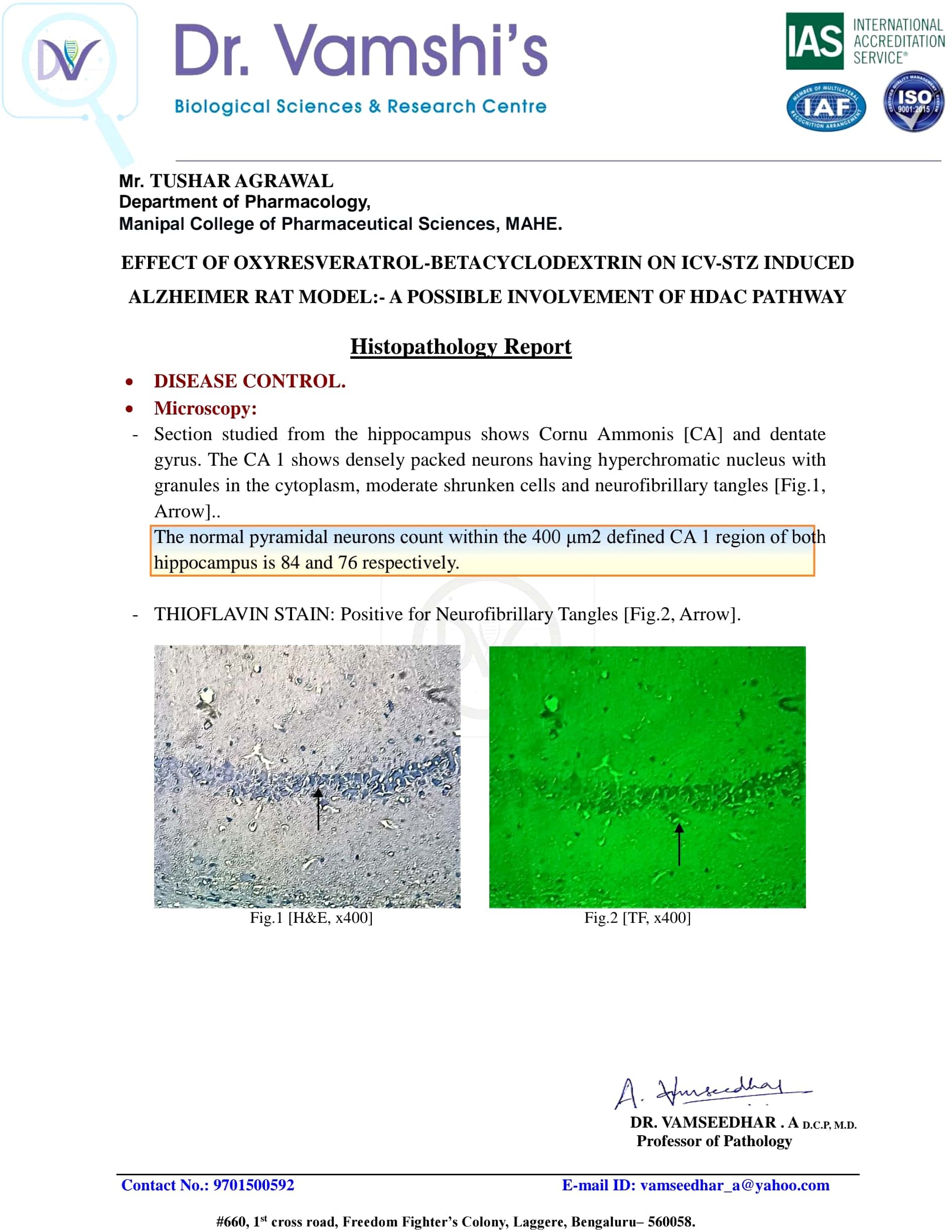


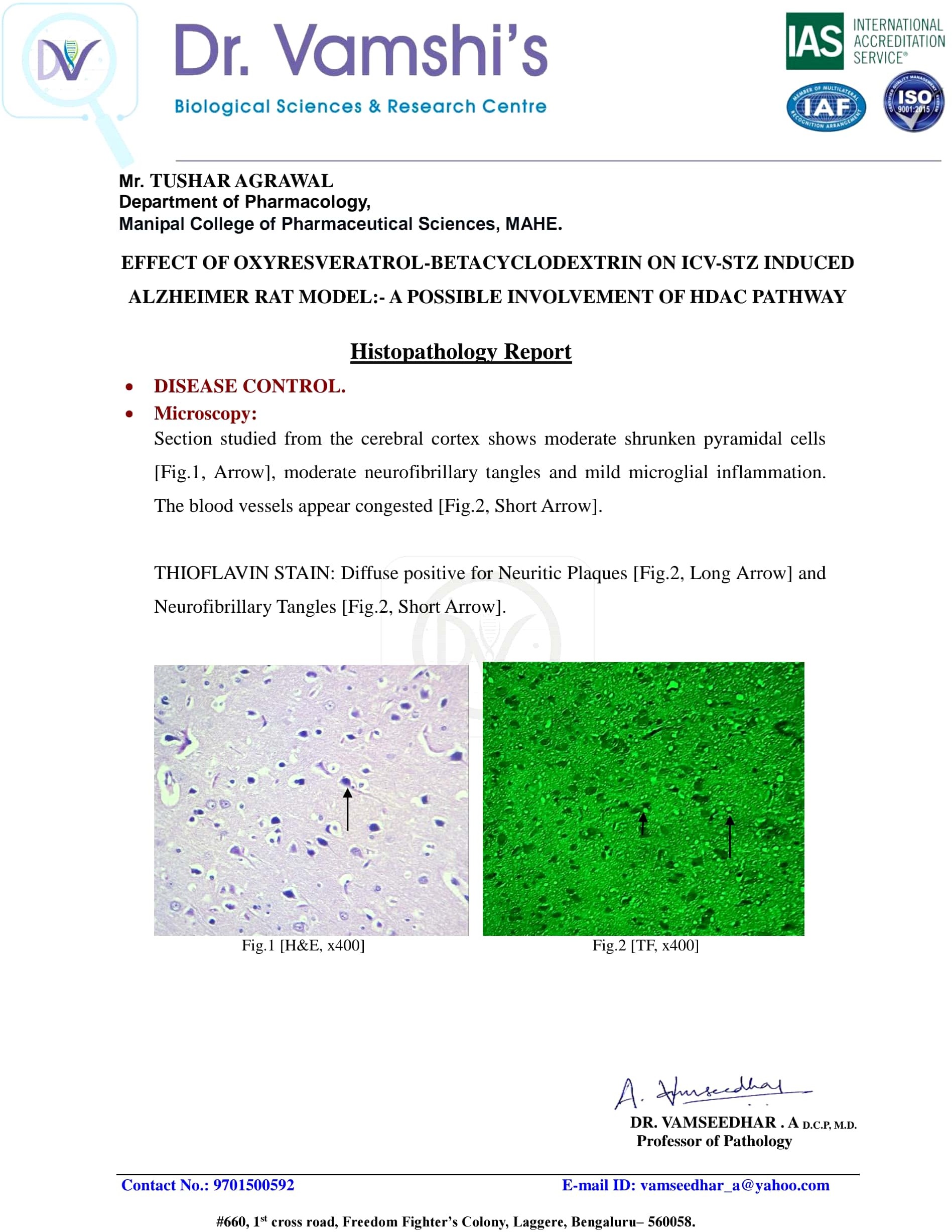


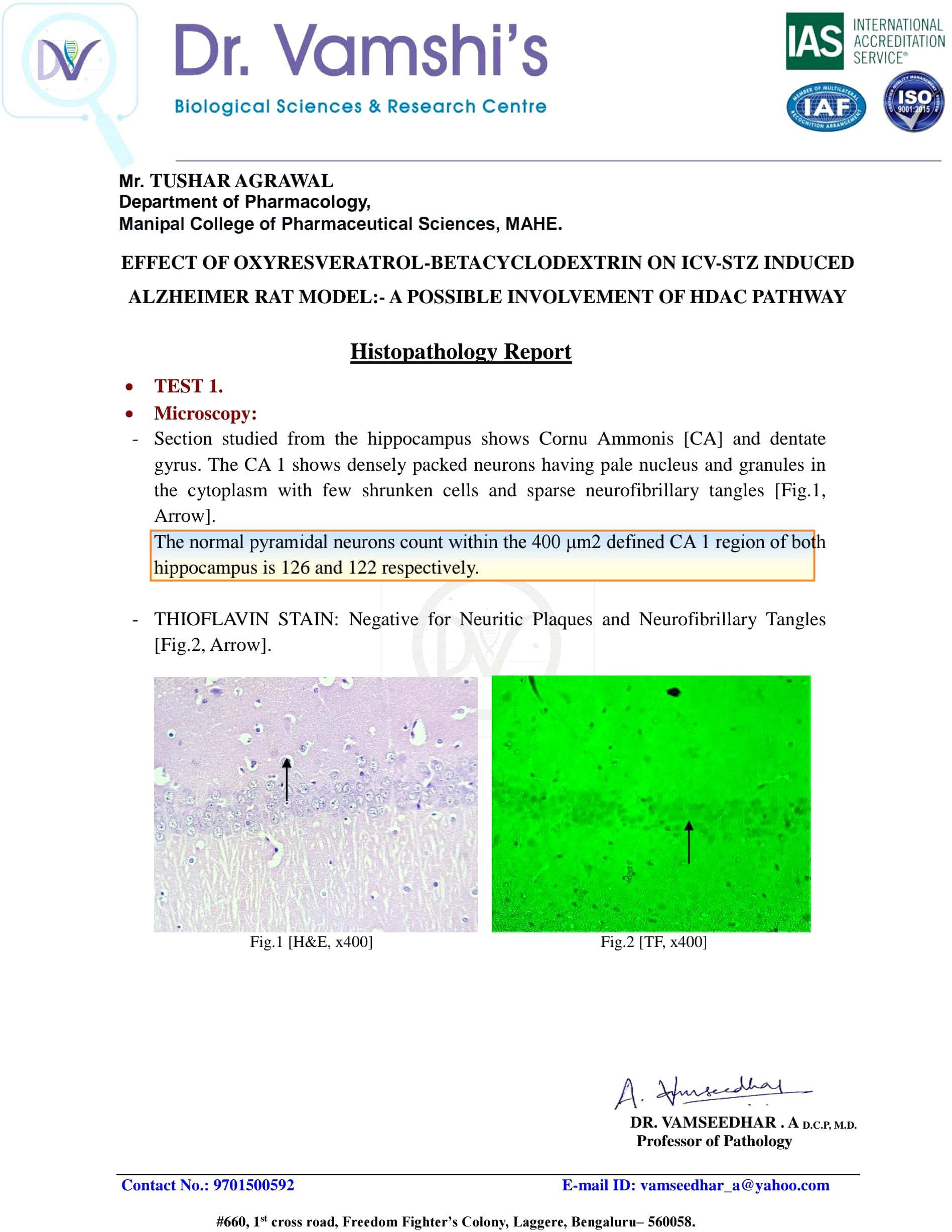


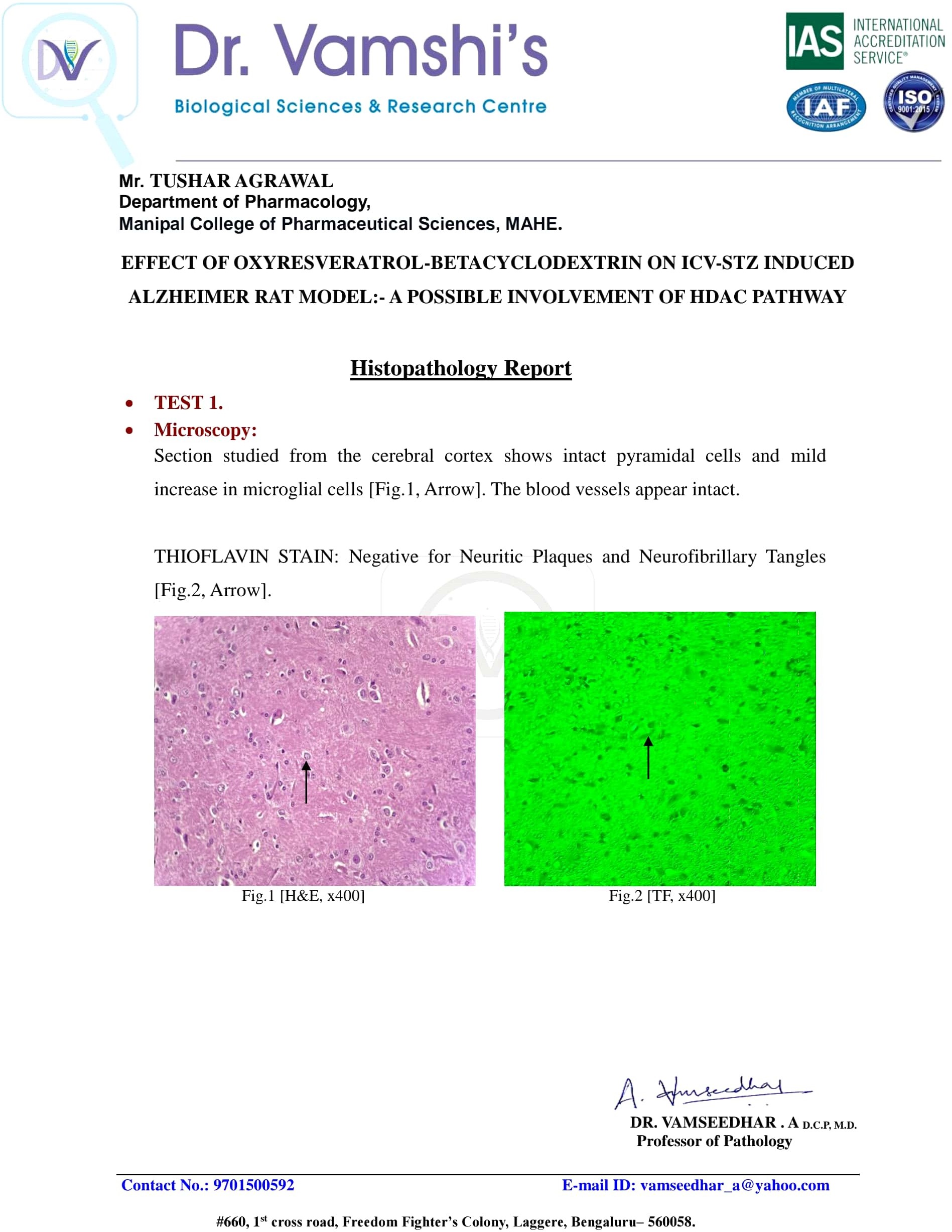


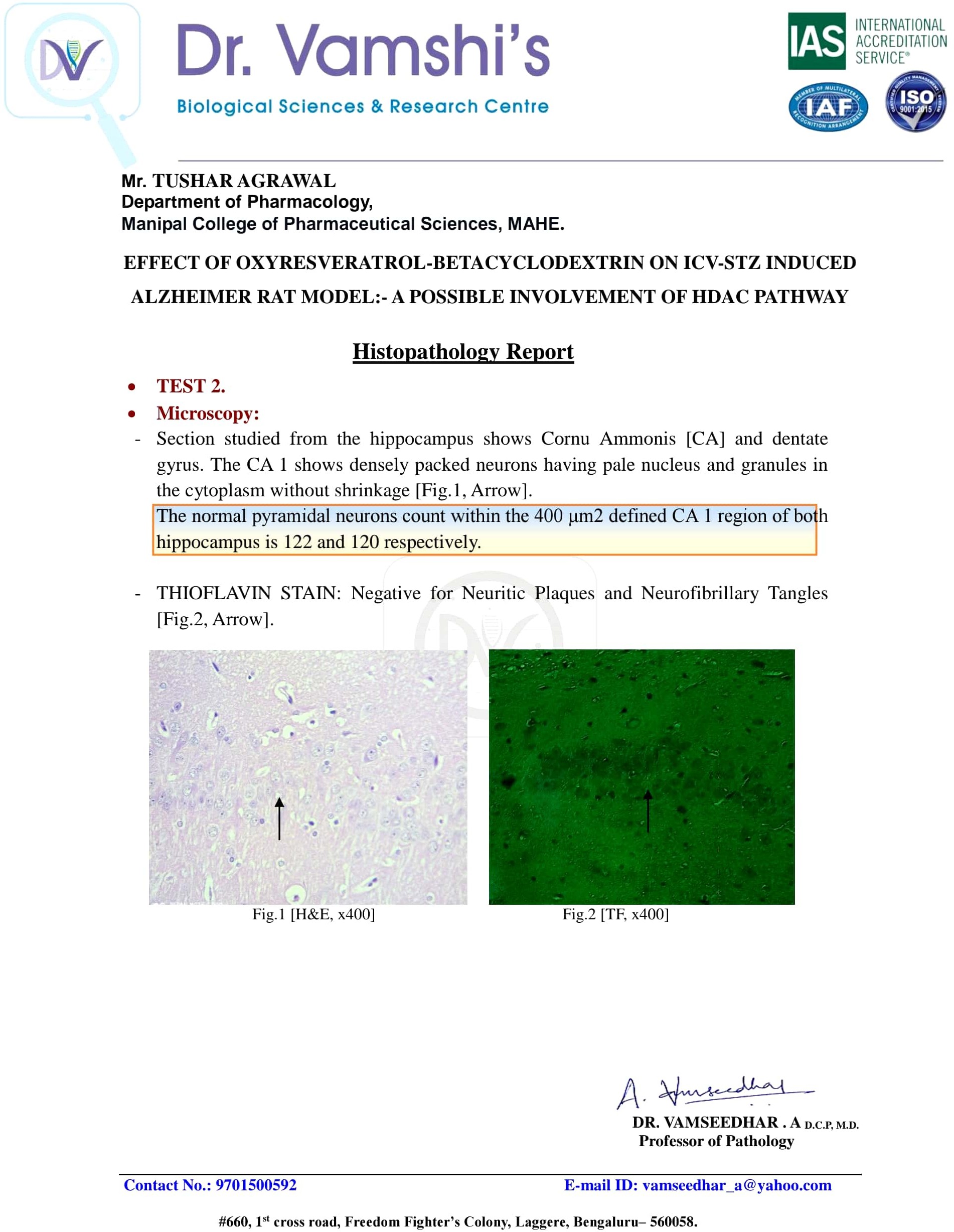


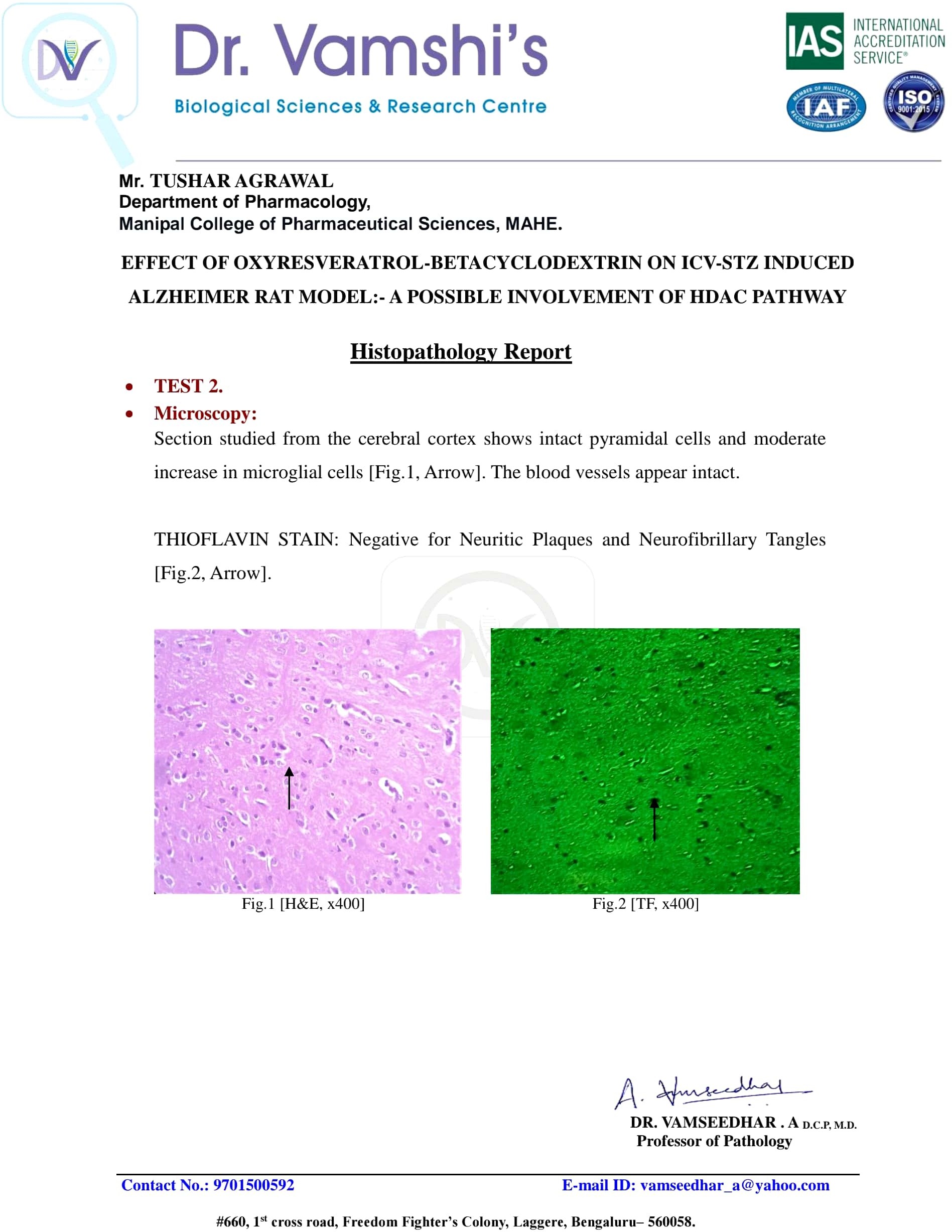

Supplement: Supplementary file 1 — Supplementary Information. [file 41598_2024_57188_MOESM1_ESM.docx]
